# Methamphetamine-mediated astrocytic pyroptosis and neuroinflammation involves miR-152–NLRP6 inflammasome signaling axis

**DOI:** 10.1016/j.redox.2025.103517

**Published:** 2025-01-25

**Authors:** Abiola Oladapo, Muthukumar Kannan, Uma Maheswari Deshetty, Seema Singh, Shilpa Buch, Palsamy Periyasamy

**Affiliations:** Department of Pharmacology and Experimental Neuroscience, University of Nebraska Medical Center, Omaha, NE, 68198-5880, USA

**Keywords:** Methamphetamine, Astrocytes, Pyroptosis, NLRP6, Neuroinflammation, miR-152

## Abstract

Methamphetamine is a widely abused drug associated with significant neuroinflammation and neurodegeneration, mainly through the activation of glial cells and neurons in the central nervous system. This study investigates the role of the astrocyte-specific NOD-like receptor family pyrin domain-containing protein 6 (NLRP6) inflammasome in methamphetamine-induced astrocytic pyroptosis and neuroinflammation. Our findings demonstrate that methamphetamine exposure induces NLRP6-dependent pyroptosis, astrocyte activation, and the release of proinflammatory cytokines in mouse primary astrocytes. Gene silencing of NLRP6 reduces methamphetamine-induced pyroptosis and proinflammatory cytokines release. We also identified miR-152 as a critical upstream regulator of NLRP6, which is downregulated in methamphetamine-exposed astrocytes. Overexpression of miR-152 decreases NLRP6 expression, mitigating methamphetamine-induced pyroptosis and inflammation. *In vivo* and *ex vivo* studies in methamphetamine-exposed mice confirmed these results and showed that methamphetamine induces anxiety-like, cognitive impairment, and depression-like behavior, further linking astrocyte-specific NLRP6 signaling to methamphetamine-induced neuroinflammation. This study highlights the potential of targeting the NLRP6 inflammasome in astrocytes as a therapeutic approach to alleviate methamphetamine-induced central nervous system pathology. Further research is warranted to explore clinical applications and identify therapeutic targets for methamphetamine-related neurological disorders.

## Highlights

• Methamphetamine induces astrocyte-specific NLRP6 inflammasome-dependent pyroptosis.
• Gene silencing of NLRP6 inhibits methamphetamine-induced pyroptosis in astrocytes.
• MiR-152 regulates NLRP6 inflammasome-mediated astrocytic pyroptosis.
• Overexpression of miR-152 protects methamphetamine-induced pyroptosis in astrocytes.

## 1 Introduction

The global prevalence of methamphetamine misuse is estimated at approximately 37 million people, with over 10 million users in the United States alone [1]. This highly addictive psychostimulant has caused substantial societal and economic burdens, with a five-fold increase in drug-related deaths over the past two decades [2]. The neurotoxic effects of methamphetamine are particularly alarming, as they target the central nervous system (CNS), resulting in cognitive dysfunction, memory impairments, anxiety, and paranoia, as well as structural brain damage [[3], [4], [5], [6]]. Methamphetamine exposure leads to pronounced changes in the CNS, including cingulate grey matter loss, hippocampal atrophy, white matter abnormalities, reduced neuronal markers, and increased glial cell activation [[7], [8], [9]]. Among glial cells, astrocytes – key players in CNS homeostasis – are critically impacted, showing dysregulated activation, migration, and proinflammatory cytokine release, which collectively contribute to neuroinflammation [[9], [10], [11], [12]]. However, the precise molecular mechanisms by which methamphetamine induces astrocyte activation and neurotoxicity remain poorly understood.

Astrocytes, the most abundant glial cell population, perform crucial roles such as maintaining the blood-brain barrier, regulating synaptic transmission, and modulating ionic balance and redox homeostasis [[13], [14], [15], [16]]. Methamphetamine exposure has been shown to stimulate astrocytic activation, marked by increased glutamate release, tumor necrosis factor (TNF) production, and calcium dysregulation [9]. Notably, human organoid studies have revealed transcriptional changes in astrocytes, including upregulation of proinflammatory cytokines, inflammasome components, and apoptosis-related genes [17]. Collectively, these findings suggest that methamphetamine-mediated astrocyte activation plays a central role in driving neuroinflammation and CNS dysfunction [[18], [19], [20]]. However, the molecular mechanisms connecting astrocyte activation, inflammasome signaling, and methamphetamine-induced cell death remain incompletely characterized.

Inflammasomes are cytosolic multiprotein complexes that function as sensors of cellular stress and danger signals, orchestrating innate immune responses through the activation of caspase-1 and the release of proinflammatory cytokines such as interleukin-1β (IL-1β) and interleukin-18 (IL-18) [[21], [22], [23], [24]]. This cascade leads to pyroptosis, a highly inflammatory form of programmed cell death characterized by membrane rupture and cytokine release [[25], [26], [27]]. While the role of the Nucleotide-binding domain Leucine-rich Repeat (NLR) containing protein 3 (NLRP3) inflammasome in neuroinflammatory and neurodegenerative diseases is well-documented [[28], [29], [30], [31]], relatively little is known about the function of the NLR containing protein 6 (NLRP6) inflammasome in the CNS. Emerging evidence highlights the involvement of NLRP6 in modulating inflammation in peripheral tissues, including the liver and intestine [[32], [33], [34], [35], [36], [37], [38], [39], [40]]. Within the CNS, NLRP6 is predominantly expressed in astrocytes and has been implicated in exacerbating neuroinflammation and pyroptosis in preclinical models of brain injury [28,41,42]. Despite these insights, the role of NLRP6 in methamphetamine-induced neuroinflammation remains unknown.

This study seeks to address this gap by investigating the role of the NLRP6 inflammasome in methamphetamine-mediated astrocytic activation and neuroinflammation. Specifically, we hypothesize that methamphetamine induces NLRP6 inflammasome activation in astrocytes, leading to pyroptosis and the release of proinflammatory cytokines, which collectively contribute to neuroinflammation. Additionally, we explore the regulatory role of the miR-152-NLRP6 axis in controlling these processes. By elucidating the molecular pathways underlying astrocyte-specific inflammasome activation, this study aims to provide critical insights into the mechanisms driving methamphetamine-induced neurotoxicity and identify potential therapeutic targets for mitigating neuroinflammation.

## 2 Materials and methods

### 2.1 Reagents

Methamphetamine hydrochloride (Cat No. M8750-5G) was procured from Sigma-Aldrich, St. Louis, MO, USA. Lipoteichoic acid (LTA; Cat No. tlrl-slta) was obtained from InvivoGen, San Diego, CA, USA. Anti-NLRP6 (Cat No. ABF29), anti-glial fibrillary acidic protein 1 (GFAP; Cat No. G3893), and anti-IL18 (Cat No. 06–1115) were purchased from Millipore-Sigma, Burlington, MA, USA. Anti-caspase-1 (Cat No. AG-20B-0042-C100) and anti-ASC, pAb (AL177) (Cat No. AG-25B-0006-C100) were sourced from AdipoGen Life Sciences, San Diego, CA, USA. Anti-IL-1β (Cat No. ab9722) and Anti-GSDMD antibody (Cat No. ab155233) were obtained from Abcam, Boston, MA, USA. The conjugated anti-β-actin-HRP (Cat No. sc-47778) was purchased from Santa Cruz Biotechnology, Dallas, TX, USA. The goat anti-mouse IgG Peroxidase-AffiniPure (H + L) (Cat No. 111-035-003) and goat anti-rabbit IgG peroxidase-conjugated AffiniPure (H + L) (Cat No. 115-035-003) were acquired from Jackson ImmunoResearch Laboratory Inc, West Grove, PA, USA. Triton® X-100 (Cat No. BP151-500), ProlongTM Gold antifade DAPI (Cat No. P36935), goat anti-mouse Alexa Fluor 488 (Cat No. A11001), and goat anti-rabbit Alexa Fluor 594 (Cat No. A11012) were purchased from Thermo Fisher Scientific, Waltham, MA, USA. Normal goat serum (Cat No. S-1000) was obtained from Vector Laboratories, Burlingame, CA, USA. The Reverse Transcription miRNA Kit (Cat No. 4366596), TaqMan® miRNA assays primer for miRNA-152 (Assay ID. 000475), TaqMan™ miRNA Control Assay – U6 snRNA primer (Assay ID. 001973), and TaqMan® Universal PCR Master Mix kit (Cat No. 4324018) were sourced from Applied Biosystems, Waltham, MA, USA. The negative control small interfering RNA (siRNA) (Cat No. 4390844), silencer select siRNA NLRP6 (Cat No. 4390771), mirVana™ miRNA mimic negative control (Cat No. 4464058), mirVana™ miRNA mimic hsa-miR-152 (Assay ID: MC12269), IL1β (assay ID: Mm00434228), IL18 (assay ID: Mm00434226), and GAPDH (assay ID: Mm99999915) were purchased from ThermoFisher Scientific, Waltham, MA, USA. Lipofectamine™ RNAiMAX reagent (Cat No. 13778150) and Opti-MEM® I Reduced Serum Media (Cat No. 31985070) were acquired from Life Technologies, Inc, Carlsbad, CA, USA. Propidium Iodide Staining Solution (Cat No. 00–6990) and Flow Cytometry Staining Buffer (Cat No. 00–4222) were purchased from Thermo Fisher Scientific, Waltham, MA, USA. Buffer A containing HEPES (Cat No. H4034), Potassium hydroxide (KOH; Cat No. P-1767), Ethylenediaminetetraacetic acid disodium salt solution (EDTA; Cat No. 03690), and Phenylmethanesulfonyl fluoride solution, (PMSF; Cat No. 93482) were sourced from Millipore Sigma, St. Louis, MO, USA. Potassium chloride (KCl; Cat No. BP3661), 40 μm Nylon mesh sterile cell strainer (Cat No. 22363547), and Magnesium dichloride (MgCl_2_; Cat No. BP214500) were purchased from Fisher Scientific, Inc, Hampton, NH, USA. Disuccinimidyl suberate (DSS; Cat No. 21555), and (3-(3-Cholamidopropyl)dimethylammonio)-1-propanesulfonate) detergent (CHAPS; Cat No. 28300) were obtained from Thermo Fisher Scientific, Waltham, MA, USA. Ethyleneglycol-bis(2-aminoethyl ether)-N, N, N1, N1-tetraacetic acid (EGTA; Cat No. 40520008–2) was sourced from Bioworld Molecular Tools for Life Science, Dublin, OH, USA. 2X SDS-PAGE sample buffer without DTT or β-mercaptoethanol (Cat No. MB-018) was purchased from Rockland Inc, Pottstown, PA, USA. Disulfiram (Cat No. HY-B0240), Necrostatin-1 (Cat No. HY-15760), and Z-VAD-FMK (Cat No. HY-16658B) were purchased from MedChemExpress, Monmouth Junction, NJ, USA.

### 2.2 Animals

Wild-type C57BL/6 mice and their age-matched counterparts (8 weeks old, both sexes) were used in this study. For *in vivo* validation experiments, we included n = 8 mice per group, and for *ex vivo* adult astrocyte experiments, we used n = 3 mice per group. All mice were housed and handled in compliance with the University of Nebraska Medical Center Institutional Animal Care and Use Committee protocol (Approval #18-030-04-FC).

### 2.3 Methamphetamine administration

For short-term methamphetamine administration, wildtype mice were acclimated to the drug using an escalating protocol [43,44]. The concentration of methamphetamine was gradually increased over five days, starting from 2 mg/kg and reaching 10 mg/kg, with the drug dissolved in sterile saline and delivered intraperitoneally. Following the escalation phase, the mice were maintained on a dose of 10 mg/kg for an additional 14 days. Untreated mice received saline injections as a control group. Behavioral assessments were conducted 1 h after the final methamphetamine or saline injection. After the behavioral experiments, the animals were anesthetized with isoflurane and euthanized by cervical dislocation. The brains were then isolated, and various brain regions, including the frontal cortex, striatum, and hippocampus, were dissected and stored at −80 °C for subsequent RNA or protein analysis. For immunohistochemistry analysis, animals were perfused with 4 % paraformaldehyde (PFA) prior to brain isolation.

### 2.4 Isolation of mouse primary astrocytes

Isolation of mouse primary astrocytes was performed with modifications to previously published methods [45,46]. Briefly, mixed mouse primary glia cultures were prepared from 1 to 3-day-old C57BL/6 newborn pups. The whole brains of the mouse pups were dissected in 1X HBSS on ice and mechanically dissociated using gauze to remove large blood vessels and membranes. The brain tissues were digested in Trypsin-EDTA and incubated at 37 °C for 10 min with intermittent agitation. The trypsin reaction was stopped with 1X FBS and filtered through a 40 μm Nylon mesh sterile cell strainer (Cat No. 22363547, Thermo Fisher Scientific, Waltham, MA, USA) to obtain a single-cell population. These cells were sedimented, resuspended, and seeded in cell culture flasks in Dulbecco's modified Eagle's medium (DMEM) supplemented with 10 % FBS, OPI media supplement, 1 % l-leucine methyl ester, and 1X penicillin/streptomycin. After 7–10 days of culturing, mouse primary astrocytes were harvested by trypsinization and seeded on cell culture plates for all experiments.

### 2.5 End-point cell death imaging

The end-point cell death analysis was monitored using the IncuCyte S3 live-cell analysis system. Mouse primary astrocytes (5x10⁵ cells/well) were seeded in 24-well cell culture plates and treated with methamphetamine (50 μM), Disulfiram (10 μM), and methamphetamine + Disulfiram. Cell death was measured using the CellTox™ Green Cytotoxicity Assay (Cat No. G8741, Promega, Madison, WI, USA) according to the manufacturer's protocol. The 24-well cell culture plate was scanned at stipulated time durations, with fluorescent and phase-contrast images acquired to measure changes in membrane integrity as a result of cell death. Cyanine dye-positive dead cells were marked with a green mask and quantified using the software package supplied with the IncuCyte imager (Version 2019BRev2).

### 2.6 Propidium iodide cell death assay

Cell death was determined by propidium iodide fluorometry using a 96-multiwell fluorescence reader (SynergyMx, Winooski, VT, USA), as previously described [47]. Briefly, mouse primary astrocytes were seeded in a 96-well cell culture plate, starved, and exposed to methamphetamine (50 μM) or LTA (5 ng/ml) for 24 h. The cells were washed twice with 1x PBS and incubated with 30 μM propidium iodide. Fluorescence intensity from each well was measured at excitation and emission wavelengths of 530 nm and 590 nm, respectively. The relative fold change in propidium iodide fluorescence intensity was calculated by normalizing the treatment groups to the control.

### 2.7 Lactate dehydrogenase (LDH) assay

The LDH cell death assay was used to measure the lactate dehydrogenase released from damaged cells using the LDH-Cytotoxicity Assay Kit (Cat No. ab65393, Abcam, Boston, MA, USA). Briefly, mouse primary astrocytes (5x10⁴ cells/well) were seeded in a 96-well cell culture plate and exposed to methamphetamine (50 μM), disulfiram, methamphetamine + disulfiram (10 μM), Necrostatin-1 (10 μM), methamphetamine + Necrostatin-1, Z-VAD-FMK (20 μM), and Meth + Z-VAD-FMK for 24 h. The cells were centrifuged at 600×*g* for 10 min to precipitate the cells, and 10 μl/well of the clear medium solution was carefully transferred into corresponding wells of an optically clear 96-well multiplate. To the transferred supernatant, 100 μl/well of LDH reaction mixture was added and incubated away from light at room temperature for 30 min. The absorbance of all samples was measured using a microtiter plate reader at 450 nm.

### 2.8 Western blotting

Western blotting was performed following standard procedures as previously described [[48], [49], [50], [51], [52]]. Briefly, mouse primary astrocytes from both the control and methamphetamine-treated groups were harvested and lysed using 200 μL of RIPA buffer (Cat No. 9806, Cell Signaling Technology, Danvers, MA, USA). The lysates were then centrifuged at 4 °C for 10 min at 12,000×*g*, and protein concentration was determined from the supernatant using the Pierce™ BCA Protein Assay Kit (Cat No. 23227, Thermo Fisher Scientific, Waltham, MA, USA), following the manufacturer's instructions. The protein lysates were prepared, and equal amounts of soluble protein from each treatment were resolved in sodium dodecyl sulfate-polyacrylamide gel electrophoresis, followed by blotting onto a polyvinylidene fluoride membrane (PVDF) (Cat No. IPVH00010, Millipore, Danvers, MA, USA). The PVDF membranes were then blocked with 5 % nonfat dry milk (in 1 × TTBS buffer) for 1 h at room temperature, followed by overnight incubation with the appropriate primary antibodies at 4 °C. Subsequently, the PVDF membranes were washed three times and incubated with the corresponding secondary antibody at room temperature for 1 h. Protein signals were visualized using Super Signal West Pico Chemiluminescent Substrate (Cat No. 34078, Thermo Fisher Scientific, Waltham, MA, USA). β-actin (Cat No. A5316, Sigma-Aldrich, St. Louis, MO, USA; 1:5000 dilution) was used as an internal control to normalize each protein band intensity. Data were represented as a relative fold change using ImageJ analysis software [53].

### 2.9 qPCR quantification of proinflammatory cytokines

The qPCR experiments were performed following a previously published protocol [[48], [49], [50], [51], [52]]. Total RNA was isolated using Quick-RNA™ MiniPrep Plus (Cat No. R1058, Zymo Research, Orange, CA, USA), according to the manufacturer's manual. Reverse transcription reactions of isolated RNA were performed using a Verso cDNA Synthesis Kit for RT-qPCR (Cat No. AB-1453/B; ThermoFisher Scientific, Inc., Pittsburgh, PA, USA). qPCRs for the determination of proinflammatory cytokine gene expression (IL1β and IL18) were completed using TaqMan® Universal PCR Master Mix, no AmpErase® UNG (Cat No. 4324018, Thermo Fisher Scientific, Waltham, MA, USA) in an Applied Biosystems® QuantStudio™ 3 Real-Time PCR System (Applied Biosystems, Grand Island, NY, USA). Each reaction was carried out in a triplicate of six independent experiments. The expression of IL1β and IL18 was normalized with *Gapdh* or *β-actin*, and the expression fold change was obtained using the 2^−ΔΔCT^ method.

### 2.10 ASC and GSDMD oligomerization assay

Human A172 astrocytoma cells seeded in 10 cm cell culture dishes (30x10^6^ cells per plate) were exposed to methamphetamine (50 μM) or LTA (5 ng/ml) for 24 h. Control and treated cells were harvested by washing twice with 1X PBS and lysed with Buffer A by syringing 30 times using a 21-G needle. The cell lysates were centrifuged at 1800×*g* for 8 min to remove the bulk nuclei, and 30 μL of the supernatants were used for western blotting analysis to determine the ASC expression. The remaining supernatant was diluted two times with Buffer A and centrifuged for 5 min at 2000×*g*. After centrifugation, the supernatant was diluted with 1 volume of CHAPS buffer, followed by centrifugation at 5000×*g* for 8 min to pellet the ASC oligomers. The pellet was resuspended in a mixture of 50 μL of CHAPS buffer and 4 mM of DSS for 30 min at room temperature with intermittent shaking. The samples were then centrifuged at 5000×*g* for 8 min, and the pellets were resuspended in 20 μL of 2 × SDS-PAGE loading buffer in nonreducing conditions for western blotting.

### 2.11 ELISA for proinflammatory cytokines

Cell culture supernatants from methamphetamine (50 μM) or LTA (5 ng/ml) exposed mouse primary astrocytes were collected and analyzed for the secretion of IL1β (Cat No. ab197742, Abcam, Boston, MA, USA) and IL18 (Cat No. ab216165, Abcam, Boston, MA, USA) using ELISA, following the manufacturer's protocol. Briefly, cell culture supernatants were collected after 24 h of treatment, centrifuged to remove debris and concentrated at 2000 g. Next, 50 μL of the sample was mixed with 50 μL of the Antibody Cocktail from the ELISA kit in a microplate well and incubated for 1 h at room temperature with gentle shaking. Each well was washed with 1X Wash Buffer PT provided in the ELISA kit and incubated with 100 μL of TMB Development Solution for 10 min in a plate shaker. Finally, 100 μL of Stop Solution was added to each well, incubated for 1 min on a plate shaker, and the optical density was recorded at 450 nm using a microplate reader.

### 2.12 GFAP and NLRP6 immunocytochemistry

Immunocytochemistry was performed on mouse primary astrocytes to determine GFAP and NLRP6 expression after treatment with methamphetamine (50 μM) or LTA (5 ng/ml) for 24 h. Mouse primary astrocytes were seeded onto sterilized glass coverslips in a 12-well plate and incubated at 37 °C for 24 h. The cells were then treated with methamphetamine or LTA (5 ng/ml) for 24 h before fixing with 4 % PFA for 20 min at room temperature. After fixation, the cells were permeabilized with 0.3 % Triton X-100 in PBS for 15 min at room temperature, followed by blocking using 10 % normal goat serum in PBS for 1 h at room temperature. Next, primary antibodies of NLRP6 (1:250) and GFAP (1:250) were used to probe the cells overnight at 4 °C. The following day, the cells were washed three times with 1X PBS and incubated for 2 h with goat anti-rabbit IgG (H + L) cross-adsorbed secondary antibody, Alexa Fluor 594 (1:1000 dilution) and goat anti-mouse IgG (H + L) cross-adsorbed secondary antibody, Alexa Fluor 488 (1:1000 dilution). After washing, the coverslips containing cells were mounted onto glass slides with ProLong Gold Antifade Reagent with DAPI. Fluorescent images were obtained using a Z1 inverted microscope on a Zeiss Observer (Carl Zeiss, Thornwood, NY, USA).

### 2.13 NLRP6 siRNA transfection

Mouse primary astrocytes were seeded into six-well plates (4.5 × 10^5^ cells per well) and transiently transfected with 30 pmol of either NLRP6 siRNA or scrambled control using Lipofectamine™ RNAiMAX (Cat No. 13778150; ThermoFisher Scientific, Inc., Pittsburgh, PA, USA) for 16 h. Next, the transfected mouse primary astrocytes were exposed to methamphetamine (50 μM; 24 h), and proteins were extracted for further analysis.

### 2.14 miRNA profiler PCR array

For the differential analysis of miRNA expression, we used the miScript™ miRNA PCR Array (Mouse Neurological Development and Disease - 331,221 MIMM-107ZA) in a 96-well plate format. Samples were harvested, and RNA was extracted from methamphetamine-treated mouse primary astrocytes. Total RNA was reverse transcribed into cDNA using the miScript II RT Kit according to Qiagen's instructions. Subsequently, qPCR was performed as previously described, employing the miScript SYBR Green PCR Kit in an Applied Biosystems® QuantStudio™ 3 Real-Time PCR System (Applied Biosystems, Grand Island, NY, USA). Normalization was conducted using specific housekeeping miRNAs included in the array.

### 2.15 miR-152 assay

The expression of miR-152 was determined using TaqMan® miR assays as described in a previous publication [52]. Briefly, total RNA isolated from methamphetamine-exposed mouse primary astrocytes was used in cDNA synthesis using TaqMan® miR Reverse Transcription kit (Cat No. 4366597; ThermoFisher Scientific, Inc., Pittsburgh, PA, USA). The expression level of miR-152 was analyzed using TaqMan Universal PCR Master mix, no AmpErase UNG (Cat No. 4324018; ThermoFisher Scientific, Inc., Pittsburgh, PA, USA), in the Applied Biosystems® QuantStudio™ 3 Real-Time PCR System (Applied Biosystems, Grand Island, NY, USA), through the comparative 2^−ΔΔCt^ method.

### 2.16 miR-152 mimic transfection

Mouse primary astrocytes were seeded into six-well plates (4.5 × 10^5^ cells per well) and transiently transfected with 30 pmol of either miR-152 mimic or miR-control using Lipofectamine™ RNAiMAX (Cat No. 13778150; ThermoFisher Scientific, Inc., Pittsburgh, PA, USA) for 16 h. Next, the transfected mouse primary astrocytes were exposed to methamphetamine (50 μM; 24 h), and total RNA and proteins were extracted for miR-152 expression and NLRP6 inflammasome signaling analysis.

### 2.17 Immunohistochemistry

Immunohistochemistry was performed following previously published protocols [52,54,55]. Brain tissue sections (5 μm) embedded in paraffin obtained from saline and methamphetamine-administered wildtype mice were baked at 55 °C overnight. Deparaffinization was carried out in xylene, followed by rehydration in a graded series of alcohol (100 %, 95 %, and 70 %). Prepared slides were washed once with PBS and then incubated in boiled Tris/EDTA buffer (pH 9) for 20 min for adequate antigen retrieval. The slides were then blocked using 10 % goat serum at room temperature for 1 h, followed by incubation with GFAP primary antibodies overnight at 4 °C. The following day, the slides were washed three times in 1X PBS and incubated for 2 h with Alexa Fluor 594 goat anti-mouse IgG (1:500) and mounted with ProLong Gold Antifade Reagent with DAPI. Fluorescent images were obtained using a Z1 inverted microscope on a Zeiss Observer (Carl Zeiss, Thornwood, NY, USA). For quantitative analysis, 5 sections per animal were analyzed, ensuring consistent sampling across comparable brain regions. The quantification of GFAP fluorescence intensity, the percentage of GFAP-labeled area, and the number of GFAP-positive cells was performed using ImageJ analysis software [53].

### 2.18 Ago2 immunoprecipitation

Ago2 immunoprecipitation was performed following the protocol provided by the manufacturer of the miR Target IP Kit (Cat No. 25500; Active Motif, Carlsbad, CA, USA). Human A172 astrocytes were seeded in 10 cm Petri dishes (2.25 × 10^6^ per dish) and transfected with either miR-152 mimic or miR-control using Lipofectamine™ RNAiMAX (Cat No. 13778150; ThermoFisher Scientific, Inc., Pittsburgh, PA, USA) for 16 h. Sample lysate was prepared using complete lysis buffer, blocked with Protein G Magnetic Beads, and labeled with 5 μg of Ago1/2/3 antibody or 25 μl of Negative Control IgG in immunoprecipitation buffer overnight at 4 °C. The samples were then treated with Proteinase K and SDS to degrade the Argonaute proteins, disrupt the antibody binding, and elute the RNA using the Phenol: Chloroform method per the manufacturer's instructions. Reverse transcription reactions of isolated RNA were performed using a Verso cDNA Synthesis Kit for RT-qPCR (Cat No. AB-1453/B; ThermoFisher Scientific, Inc., Pittsburgh, PA, USA). qPCRs were used to determine the enrichment level of NLRP6 in the total and Ago2-immunoprecipitated cDNA samples.

### 2.19 Dual luciferase reporter assay

The dual luciferase reporter assay was conducted to validate the binding site of miR-152–3p in the 3′-untranslated region (3′-UTR) of NLRP6, with modifications based on established methods [48,54]. HEK293T cells were seeded in 96-well plates and co-transfected with target plasmids (e.g., pmirGLO-NLRP6 3′-UTR-miR-152-target or pmirGLO-NLRP6 3′-UTR-miR-152-target-mutant) and miR-152 mimic or miR control at a 10:1 M ratio. After 24 h, luciferase activity was measured using the manufacturer's protocol (Cat No. E1910; Promega Corporation, Madison, WI, USA). Renilla luciferase activity was normalized to firefly luciferase and expressed as a percentage relative to the control.

### 2.20 Flow cytometry

Cells were washed twice with Flow Cytometry Staining Buffer at 1000g for 5 min. The pellets were resuspended in 1 ml of Flow Cytometry Staining Buffer. Propidium Iodide Staining Solution was added and incubated for 5–15 min on ice. The samples were analyzed by flow cytometry.

### 2.21 Ex vivo adult mouse astrocyte isolation

Adult astrocytes were isolated from both saline- and methamphetamine-administered wild-type mice (8 weeks old, male) using the Adult Brain Dissociation Kit (Cat No. 130-107-677, Miltenyi Biotec Inc., Auburn, CA, USA) according to the manufacturer's protocol. Mice were sacrificed at the end of the saline or methamphetamine administration, and their brains were extracted and immediately washed in cold D-PBS. The brains were sectioned into approximately eight sagittal slices using a sterile scalpel. The brain slices were transferred into a C tube containing enzyme mix 1 and enzyme mix 2 from the dissociation kit. The C tube was mounted upside-down onto the sleeve of a gentleMACS Octo Dissociator with Heaters and processed using the appropriate dissociation program to fully homogenize the brain tissue. The homogenized samples were briefly centrifuged and passed through a series of SmartStrainers (70 μm, 40 μm, and 30 μm) to create a single-cell suspension. The cell suspension was centrifuged at 300×*g* for 10 min at 4 °C. Debris and red blood cells were removed using the debris removal solution and red blood cell removal solution, as instructed by the manufacturer. Cell counts were performed using an Invitrogen Countess II Automated Cell Counter (Cat No. AMQAX1000, ThermoFisher Scientific, Singapore). Following another round of centrifugation at 300×*g* for 10 min at 4 °C, the resulting cell pellets were resuspended in Buffer A (1X PBS, 0.5 % BSA) and blocked with FcR Blocking Reagent for 15 min at 4 °C. Astrocytes were labeled with Anti-ACSA-2 MicroBeads (Miltenyi Biotec Inc.) and incubated at 4 °C for 15 min. The labeled cells were washed and passed through an LS column attached to a magnetic separator to deplete non-astrocytic cells. The ACSA-2-positive magnetically labeled cells were flushed out using a plunger, yielding an astrocyte-enriched cell fraction. The purity of the isolated astrocytes was determined by flow cytometry using fluorochrome-conjugated ACSA-2 antibodies. The remaining fraction of isolated cells was used for downstream analyses, including the assessment of NLRP6 inflammasome signaling and astrocyte activation.

### 2.22 Behavioral tests

Behavioral experiments were conducted over two consecutive days. On day 13 of methamphetamine administration, the Open Field Test was performed first and also served as an acclimatization period for the Novel Object Discrimination Test, which was conducted later the same day. On day 14, the Y-Maze Test was conducted in the morning, followed by overnight food deprivation to prepare for the Sucrose Preference Test. For the Sucrose Preference Test, mice were provided with two pre-weighed bottles – one containing filtered tap water and the other a 1 % sucrose solution – for 12 h in the evening.

a. *Open Field Test:* Mice were gently placed by the tail, facing the far corner of a 50 cm × 50 cm standard apparatus, and allowed to explore for 10 min. Their movements were monitored using a video tracking system. This protocol was adapted from the operational procedures of the UNMC Animal Behavioral Core for mice. Parameters measured included total mobility, the number of entries into the center zone, time spent in the center zone, average distance from the center zone, average speed in the center zone, and path efficiency.
b. *Y-Maze Test:* The spontaneous alternation protocol was employed. Mice were placed at the base of arm 1 of the maze, facing away from the other arms, and encouraged to choose arm 2 followed by arm 3 during a pretrial training period lasting 60 s. Afterward, the mouse was removed and allowed to alternate freely in the Y-maze for 8 min, with movement tracked by a video system. Parameters measured included immobility, total freezing episodes, the number of 123/321 sequences, average time to complete the 123/321 sequence, and percentage of spontaneous alternations. The percentage of spontaneous alternation was calculated as: ([Number of alternate explored arms/Total number of arm entries – 2]) × 100 [56].
c. *Novel Object Recognition Test:* Following a 10-min acclimatization period, mice underwent two training sessions, each lasting 5 min, during which they were exposed to two identical objects. The training sessions were separated by a 1-h interval. Ten minutes after the second training session, the mice were exposed to one familiar object and one novel object for 5 min to assess short-term memory. The Discrimination Index (DI), was calculated as the difference in exploration time between the novel and familiar object, divided by the sum of time exploration of the novel and familiar objects [57].
d. *Sucrose Preference Test:* This experiment consisted of two phases: fasting and testing. Mice were fasted overnight and then presented with two pre-weighed drinking bottles, one containing filtered tap water and the other containing a 1 % sucrose solution. Saline- and methamphetamine-administered mice were tested under these conditions for 12 h. After the test period, the bottles were reweighed, and the difference in weight was used to evaluate depression-like behavior in the mice.

### 2.23 Statistical analysis

All values were expressed as mean ± SEM and an appropriate statistical significance level was chosen based on the experimental strategy using GraphPad Prism version 10. For multiple group comparisons, nonparametric Kruskal–Wallis one-way ANOVA followed by Dunn's post hoc test was employed, and for two group comparisons, the Wilcoxon matched-pairs signed-rank test was employed. Unpaired Student's t-test was used to compare saline and methamphetamine-administered groups for the *in vivo* experiments. Values were considered statistically significant when P < 0.05.

## 3 Results

### 3.1 Methamphetamine exposure induced cellular activation, NLRP6 inflammasome activation, and pyroptosis in mouse primary astrocytes

**Fig. 1:**
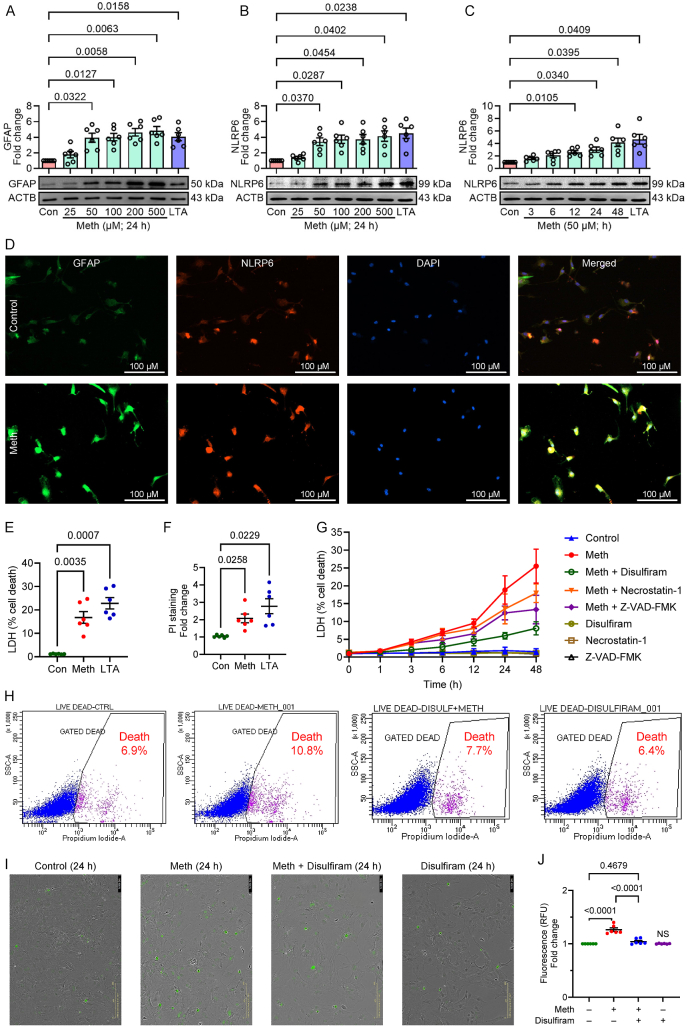
Methamphetamine exposure induces cellular activation, NLRP6 expression, and cell death in mouse primary astrocytes. (A) Representative Western blot showing dose-dependent expression of GFAP in methamphetamine-exposed mouse primary astrocytes. Representative Western blot depicting dose-dependent (B), and time-dependent (C) NLRP6 expression in methamphetamine-exposed mouse primary astrocytes. (D) Representative fluorescene microscopy images displaying dual immunostaining of GFAP and NLRP6 in methamphetamine-exposed mouse primary astrocytes (magnification: 20x, scale bar: 100 μm). Percentage of cell death in mouse primary astrocytes exposed to methamphetamine and LTA, as measured by the LDH assay (E), and relative propidium iodide intensity (F). (G) LDH assay of percentage cell death in mouse primary astrocytes exposed to methamphetamine in the presence of disulfiram (10 μM), Necrostatin 1 (10 μM), and Z-VAD-FMK (20 μM). (H) Propidium iodide flow cytometry, and (I) CellTox™ Green Cytotoxicity Assay using the Incucyte imager and quantification (J) of mouse primary astrocytes exposed to methamphetamine in the presence of disulfiram (10 μM). Scale bar: 200 μm. β-actin was used as an internal control for all experiments. Data are presented as mean ± SEM from six independent experimental replicates. LTA (5 ng/ml) was used as a positive control. Statistical significance was determined using the nonparametric Kruskal–Wallis one-way ANOVA followed by Dunn's post hoc test. NS: not significant.

Given that NLRP6 is abundantly expressed in astrocytes and plays a critical role in cellular activation and brain injury [58], this study was aimed at understanding the molecular mechanism(s) by which methamphetamine-induced NLRP6 inflammasome signaling and subsequent cellular activation in mouse primary astrocytes. To investigate methamphetamine-mediated astrocyte activation and NLRP6 inflammasome induction, mouse primary astrocytes were exposed to varying doses of methamphetamine (25–500 μM) for 24 h, followed by assessing the expression of GFAP (astrocyte activation marker) and NLRP6 protein by western blotting. As shown in Fig. 1A and B, there was a dose-dependent upregulation of GFAP and NLRP6 in methamphetamine-exposed astrocytes. Based on previously reported studies on methamphetamine tissue compartmentalization and blood concentrations [5,[59], [60], [61]] and based on our observations in Fig. 1A and B, a concentration of 50 μM was chosen for subsequent experiments. Next, a time-course experiment was performed in which mouse primary astrocytes were exposed to 50 μM methamphetamine for various time points (0, 3, 6, 12, 24, and 48 h). As shown in Fig. 1C, methamphetamine exposure to mouse primary astrocytes increased NLRP6 expression in a time-dependent manner. LTA at 5 ng/ml was used as a positive control based on another independent study that demonstrated specific binding between NLRP6 and LTA [62]. Additionally, LTA treatment (5 ng/ml for 24 h) of astrocytes was also shown to increase the expression of GFAP (Fig. 1A) as well as NLRP6 (Fig. 1B and C). A significant increase in NLRP6 mRNA expression was also observed in methamphetamine-exposed astrocytes (Fig. S1A). The findings were further validated by assessing the localization and expression levels of GFAP and NLRP6 in methamphetamine-exposed astrocytes (50 μM for 24 h) using dual immunostaining. As shown in Fig. 1D, the immunostaining revealed increased mean fluorescence intensity of both GFAP and NLRP6 in methamphetamine-exposed astrocytes compared to the control cells, thereby implicating heightened cellular activation and expression of NLRP6 in response to methamphetamine exposure. To further differentiate the involvement of canonical and non-canonical inflammasome proteins, the expression levels of NLRP6, NLRP3, and AIM2 were also determined in methamphetamine-exposed astrocytes. It was found that while NLRP6 was significantly upregulated, the expression of NLRP3 and AIM2 remained unchanged, thus highlighting the role of NLRP6 inflammasome in methamphetamine-exposed mouse primary astrocytes (Figs. S1B–C). We also sought to determine the expression levels of NLRP6, along with NLRP3 and AIM2, in microglia exposed to methamphetamine. For this purpose, we used the mouse SIM-A9 microglial cell line. Our results demonstrated increased expression of NLRP3 and AIM2, as well as the microglial activation marker CD11b, while NLRP6 expression remained unchanged in methamphetamine-exposed SIM-A9 microglial cells (Figs. S1D and E). These findings suggest that methamphetamine-induced neuroinflammation in microglia may proceed via alternative pathways that are independent of NLRP6.

Next, methamphetamine-mediated cell death was assessed using LDH and propidium iodide fluorescence assays to determine overall cell death caused by methamphetamine. A significant increase in the percentage of cell death (Fig. 1E) and a relative fold change in propidium iodide staining intensity (Fig. 1F) was observed in methamphetamine-exposed astrocytes. Next, the contributions of different programmed cell death pathways were assessed using disulfiram (10 μM, pyroptosis inhibitor), necrostatin-1 (10 μM, necroptosis inhibitor), and Z-VAD-FMK (20 μM, pan-caspase inhibitor) in LDH assays [23,63,64]. All inhibitors showed protection against methamphetamine-induced astrocyte cell death, with disulfiram having the maximum efficacy (Fig. 1G), further confirming pyroptotic cell death in these cells.

To further validate methamphetamine-mediated pyroptotic cell death in mouse primary astrocytes, propidium iodide flow cytometry, and the CellTox™ Green cytotoxicity assays were performed. The increased number of propidium iodide-positive cells induced by methamphetamine was reduced by pretreatment of the cells with disulfiram (Fig. 1H). Real-time cell death imaging using the CellTox™ Green cytotoxicity assay also showed increased numbers of cyanine dye-positive dead cells in methamphetamine-exposed mouse primary astrocytes, which were significantly decreased with disulfiram pretreatment (Fig. 1I and J), thus confirming the involvement of pyroptotic cell death among other programmed inflammatory cell death pathways.

### 3.2 Methamphetamine exposure induced the expression of the NLRP6 inflammasome signaling mediators in mouse primary astrocytes

**Fig. 2:**
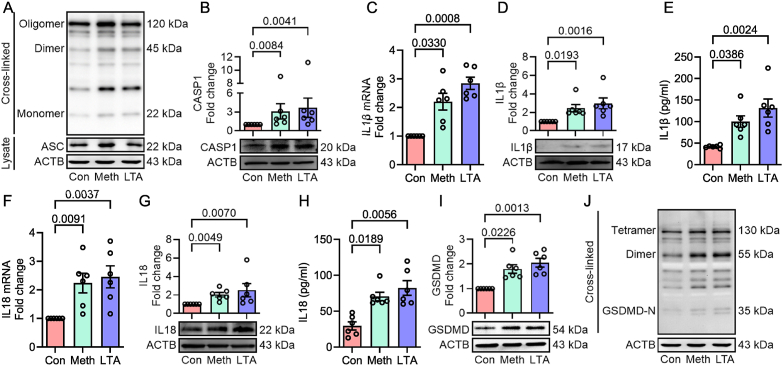
Methamphetamine induces inflammasome activation and pyroptosis in mouse primary astrocytes. (A) Representative Western blot analysis of ASC in crosslinked pellet (upper panel) and cell lysates (lower panel) with quantification in methamphetamine-exposed human A172 astrocytes (N = 3). (B) Representative Western blot analysis showing the expression of cleaved caspase-1 in methamphetamine-exposed mouse primary astrocytes. qPCR analysis showing the mRNA expression of IL1β (C), representative Western blot analysis showing the protein expression of matured IL1β (D), ELISA showing the IL1β protein levels (E) in methamphetamine-exposed mouse primary astrocytes. qPCR analysis showing the mRNA expression of IL18 (F), representative Western blot analysis showing the protein expression of matured IL18 (G), ELISA showing the IL18 protein levels (H) in methamphetamine-exposed mouse primary astrocytes. Representative Western blot analysis showing the expression of GSDMD in cell lysates (I) in methamphetamine-exposed mouse primary astrocytes and crosslinked pellet (J) in methamphetamine-exposed human A172 astrocytes (N = 3). β-actin was used as an internal control for all experiments. Data are presented as mean ± SEM from six independent experimental replicates. LTA (5 ng/ml) was used as a positive control. Statistical significance was determined using the nonparametric Kruskal–Wallis one-way ANOVA followed by Dunn's post hoc test.

To investigate the expression profile of NLRP6 inflammasome signaling downstream mediators in methamphetamine-exposed astrocytes, ASC oligomerization, the cleavage levels of caspase-1, Gasdermin D (GSDMD), and the induction of IL1β and IL18 were determined in cells exposed to methamphetamine (50 μM) for 24 h. As shown in Fig. 2A, methamphetamine exposure caused the formation of ASC oligomers, dimers, and monomers in human A172 astrocytes, thus confirming the formation of the NLRP6 inflammasome complex and its activation in these cells. Moreover, methamphetamine exposure also increased the expression of ASC in the mouse primary astrocytes (Fig. S1F). Next, the key downstream mediators of NLRP6 inflammasome activation, including caspase-1 cleavage, the maturation, and release of IL1β and IL18, and GSDMD-dependent pyroptosis, were determined in methamphetamine (50 μM; 24 h) exposed mouse primary astrocytes with LTA (5 ng/ml; 24 h) as positive control. As shown in Fig. 2B–H, exposure of mouse primary astrocytes to methamphetamine significantly increased the cleavage of caspase-1 (Fig. 2B), as well as the mRNA and protein expression of IL1β (Fig. 2C–E) and IL18 (Fig. 2F–H) compared to control cells. Furthermore, methamphetamine exposure significantly increased the protein expression of GSDMD (Fig. 2I) in mouse primary astrocytes and GSDMD oligomerized components (Fig. 2J) in human A172 astrocytes compared to control cells.

### 3.3 NLRP6 inflammasome activation is essential for methamphetamine-mediated pyroptosis and cellular activation

**Fig. 3:**
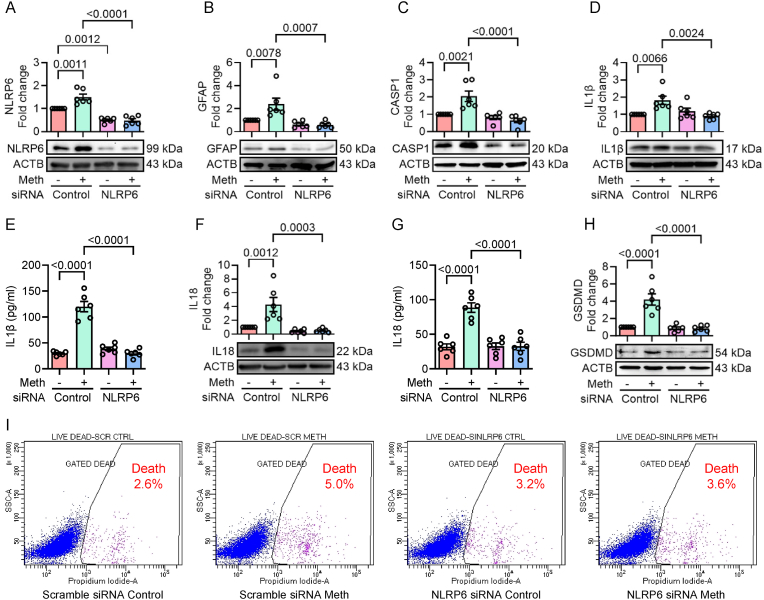
NLRP6 is critical for methamphetamine-mediated activation of astrocytic pyroptosis. Representative Western blot analysis showing the expression of NLRP6 (A), GFAP (B), and cleaved caspase-1 (C) in mouse primary astrocytes transfected with NLRP6 siRNA and exposed with methamphetamine (50 μM) for 24 h. Representative Western blot analysis showing the protein expression of IL1β (D) and ELISA showing the IL1β protein levels (E) in mouse primary astrocytes transfected with NLRP6 siRNA and exposed with methamphetamine (50 μM) for 24 h. Representative Western blot analysis showing the protein expression of IL18 (F) and ELISA showing the IL18 protein levels (G) in mouse primary astrocytes transfected with NLRP6 siRNA and exposed with methamphetamine (50 μM) for 24 h. Representative Western blot analysis showing the expression of GSDMD (H) in mouse primary astrocytes transfected with NLRP6 siRNA and exposed with methamphetamine (50 μM) for 24 h. (I) Flow cytometry analysis of propidium iodide-positive cells, indicating pyroptosis, in mouse primary astrocytes transfected with NLRP6 siRNA and administered with methamphetamine (50 μM) for 24 h. β-actin was used as an internal control for all experiments, and the data are presented as mean ± SEM. Statistical significance was determined using the nonparametric Kruskal–Wallis one-way ANOVA followed by Dunn's post hoc test.

To investigate the involvement of the NLRP6 inflammasome in methamphetamine-mediated pyroptosis and cellular activation, a gene silencing approach targeting NLRP6 was employed. Mouse primary astrocytes were transiently transfected with either scrambled siRNA or NLRP6 siRNA, followed by methamphetamine exposure (50 μM) for 24 h. As shown in Fig. 3A, a successful knockdown of NLRP6 protein expression was confirmed in both control and methamphetamine-exposed NLRP6 siRNA-transfected groups of cells compared to the scrambled groups. Furthermore, the protein expression of GFAP, an astrocyte activation marker, and downstream mediators of NLRP6 inflammasome signaling, such as cleaved caspase-1, GSDMD, and proinflammatory cytokines IL1β and IL18, were investigated. Silencing of NLRP6 significantly attenuated the methamphetamine-mediated increased expression of GFAP (Fig. 3B), caspase-1 (Fig. 3C), the maturation, and release of IL1β (Fig. 3D–E) and IL18 (Fig. 3F–G), and GSDMD (Fig. 3H) compared with control cells. Similarly, and as expected, the increased numbers of propidium iodide-positive cells (cell death) were significantly decreased in the NLRP6 siRNA-transfected cells following methamphetamine exposure (Fig. 3I).

### 3.4 miR-152 targets the 3′-UTR of NLRP6 in mouse primary astrocytes

**Fig. 4:**
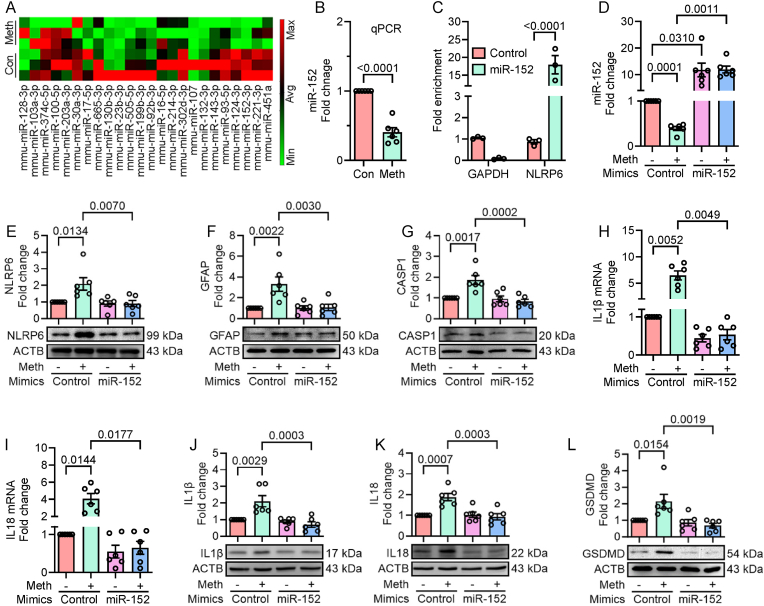
miR-152 targets the 3′-UTR of NLRP6 in mouse primary astrocytes. (A) Heatmap showing the differential expression of miRs in mouse primary astrocytes exposed to methamphetamine (50 μM; 24 h). (B) qPCR analysis showing the expression of miR-152 in methamphetamine-exposed mouse primary astrocytes (50 μM; 24 h), with U6 used as an endogenous control. (C) miRNA target immunoprecipitation assay confirming increased enrichment of NLRP6 in miR-152 mimic overexpressed human A172 astrocytes. (D) qPCR analysis showing the transfection efficiency of miR-152 in methamphetamine-exposed mouse primary astrocytes (50 μM; 24 h), with U6 used as an endogenous control. Represntative Western blot analysis showing the expression of NLRP6 (E), GFAP (F), and cleaved caspase-1 (G) in mouse primary astrocytes transfected with miR-152 and exposed with methamphetamine (50 μM; 24 h). qPCR analysis showing the mRNA expression of IL1β (H) and IL18 (I) in mouse primary astrocytes transfected with miR-152 and exposed with methamphetamine (50 μM; 24 h). Represntative Western blot analysis showing the protein expression of IL1β (J), IL18 (K), and GSDMD (L) in mouse primary astrocytes transfected with miR-152 and exposed with methamphetamine (50 μM; 24 h). β-actin was used as an endogenous control for all experiments. Data are presented as mean ± SEM from six independent experimental replicates. Statistical significance was determined using the nonparametric Kruskal–Wallis one-way ANOVA followed by Dunn's post hoc test.

Thus far, the results have consistently shown that the NLRP6 inflammasome is a mediator in methamphetamine-mediated pyroptosis in mouse primary astrocytes. To determine the upstream regulator responsible for NLRP6 inflammasome-dependent pyroptosis in methamphetamine-exposed mouse primary astrocytes, the expression profiles of miRs in methamphetamine-exposed mouse primary astrocytes were determined using the miScript miR PCR Array for Mouse Neurological Development and Disease. As shown in Fig. 4A, several miRs were differentially expressed in response to methamphetamine (50 μM; 24 h) exposure. Among the downregulated miRNAs, miR-152 expression was most significantly reduced, followed by miR-124, miR-132, miR-221, and miR-451a. Subsequent validation using qPCR confirmed the decreased expression of miR-152 in methamphetamine-exposed mouse primary astrocytes (50 μM; 24 h), as shown in Fig. 4B. To confirm the direct binding and regulation of miR-152 with the 3′-UTR of NLRP6, Argonaute immunoprecipitation was performed using a miRNA target validation kit following the manufacturer's instructions. As shown in Fig. 4C, this assay demonstrated significant enrichment of NLRP6 mRNA bound to Argonaute protein in cells overexpressing the miR-152 mimic, confirming NLRP6 as a direct target of miR-152. To further validate this interaction, a dual luciferase assay was conducted. Co-transfection of HEK293T cells with a pmirGLO dual luciferase reporter plasmid containing the wild-type NLRP6 3′-UTR and miR-152 mimic significantly reduced luciferase activity (Fig. S2). In contrast, no such effect was observed when a plasmid containing a mutated NLRP6 3′-UTR was co-transfected with miR-152 mimic (Fig. S2).

Next, to determine the regulatory role of miR-152 in methamphetamine-mediated NLRP6 inflammasome-dependent pyroptosis in mouse primary astrocytes, cells were transfected with either a miR-control or miR-152 mimic, followed by methamphetamine (50 μM; 24 h) exposure. The expression levels of miR-152, NLRP6, GFAP, caspase 1, IL1β, IL18, and GSDMD were subsequently assessed. As shown in Fig. 4D, qPCR analysis confirmed the transfection efficiency of miR-152, showing significantly increased expression of miR-152 in both the control and methamphetamine-exposed mouse primary astrocytes overexpressing miR-152. Methamphetamine-mediated increased expression of NLRP6 (Fig. 4E), GFAP (Fig. 4F), and caspase 1 (Fig. 4G) was also significantly reversed in miR-152 overexpressed mouse primary astrocytes exposed to methamphetamine. Additionally, the increased expression of IL1β mRNA (Fig. 4H), IL18 mRNA (Fig. 4I), mature IL1β (Fig. 4J), mature IL18 (Fig. 4K), and GSDMD (Fig. 4L) in methamphetamine-exposed mouse primary astrocytes was significantly downregulated in the miR-152 overexpressed methamphetamine-exposed group.

### 3.5 *In vivo* validation of NLRP6 inflammasome signaling mediated pyroptosis in the brain tissues of methamphetamine-administered mice

**Fig. 5:**
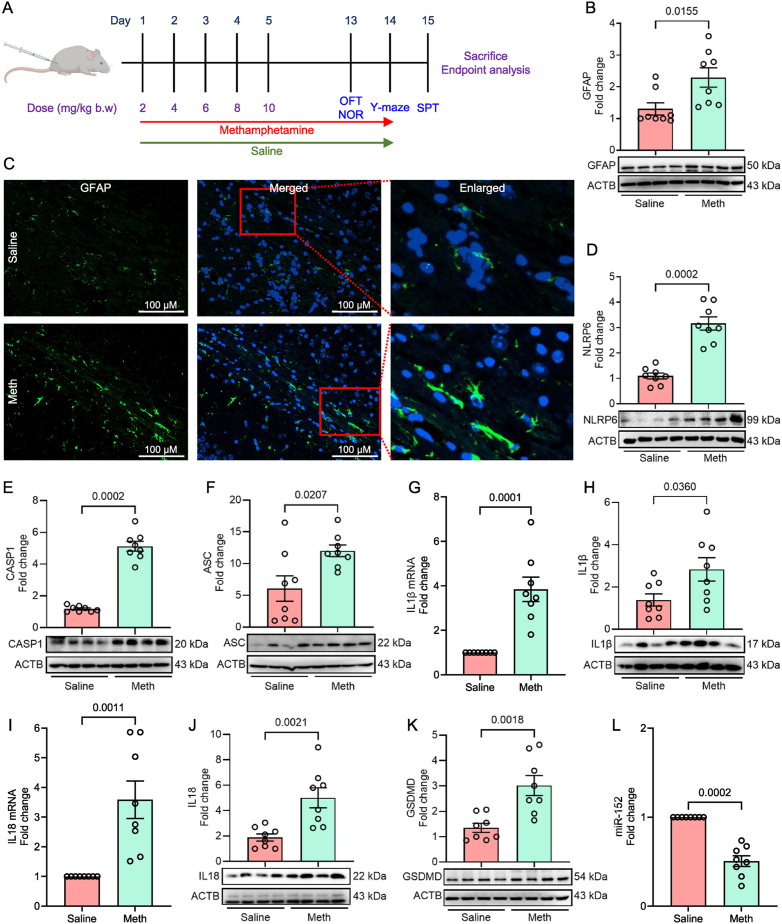
Methamphetamine-induced NLRP6 inflammasome signaling and astrocyte activation *in vivo*. (A) Schematic outline of the experimental protocols for administering escalating doses of methamphetamine and saline to wildtype mice. (B) Representative Western blot and (C) immunohistochemistry images showing the expression of GFAP in the hippocampus sections of wildtype mice administered with methamphetamine or saline (Scale bar: 100 μm). Representative Western blot images showing the expression of NLRP6 (D), cleaved caspase-1 (E), and ASC (F) in the hippocampus of wildtype mice administered with methamphetamine or saline. qPCR analysis showing the mRNA expression of IL1β (G) and representative Western blot showing the protein expression of IL1β (H) in the hippocampus of wildtype mice administered with methamphetamine or saline. qPCR analysis showing the mRNA expression of IL18 (I) and representative Western blot showing the protein expression of IL18 (J) and GSDMD (K) in the hippocampus of wildtype mice administered with methamphetamine or saline. (L) qPCR analysis showing the expression of miR-152 in hippocampus of wildtype mice administered with methamphetamine or saline. Statistical significance was determined using an unpaired Student's t-test. Representative Western blot images are shown for n = 4 per group, as it was not feasible to run all samples on a single blot. However, quantification was performed for n = 8 per group using two separate rounds of western blotting. Abbreviations: OFT: open-field test; NOR: novel object recognition; SPT: sucrose preference test.

To validate the *in vitro* findings, the critical mediators of NLRP6 inflammasome activation were measured *in vivo* in the brain tissue of saline and methamphetamine-administered mice. Eight-week-old (both sexes) mice were used in this study. The experimental setup for determining methamphetamine-induced NLRP6 inflammasome signaling *in vivo* is shown in Fig. 5A. The expression levels of GFAP, NLRP6, and downstream signaling mediators such as caspase-1, ASC, IL1β, IL18, GSDMD, and miR-152 were assessed in the frontal cortex, striatum, and hippocampus brain regions of methamphetamine-administered mice. These regions were selected due to their involvement in memory, emotion, and reward pathways, which are essential for methamphetamine-induced structural alteration and cognitive impairment [7,65,66].

The extent of astrocyte activation was determined using western blotting and immunohistochemical staining for GFAP. The results indicated that methamphetamine-administered mice exhibited significantly increased GFAP expression, as demonstrated by western blotting (Fig. 5B) and enhanced GFAP immunoreactivity (Fig. 5C; Figs. S3A–C) in the hippocampus compared to saline-administered controls. Furthermore, methamphetamine-administered mice displayed increased GFAP immunoreactivity in the hippocampal subregions, including the dentate gyrus (DG) and Cornu Ammonis (CA1, CA2, CA3, and CA4; Figs. S4A–E).

As shown in Fig. 5D–F, the expression levels of NLRP6 (Fig. 5D), caspase 1 (Fig. 5E), and ASC (Fig. 5F) were also significantly increased in the hippocampus of methamphetamine-administered mice compared to the saline group, indicating the formation of the NLRP6 inflammasome complex. Similarly, expression levels of IL1β mRNA (Fig. 5G), mature IL1β protein (Fig. 5H), IL18 mRNA (Fig. 5I), and mature IL18 protein (Fig. 5J) were also significantly increased in the hippocampus regions of methamphetamine-administered mice compared to the saline group. Additionally, the expression of GSDMD was significantly upregulated in the hippocampus of methamphetamine-administered mice compared to the saline group (Fig. 5K). Furthermore, qPCR analysis revealed decreased expression of miR-152 in the hippocampus (Fig. 5L) of methamphetamine-administered mice compared to the saline group. Similar results were observed in the frontal cortex (Figs. S5A–I) and striatum (Figs. S6A–I) of methamphetamine-administered mice compared to the saline group.

### 3.6 Behavioral deficits in methamphetamine-administered wildtype mice

**Fig. 6:**
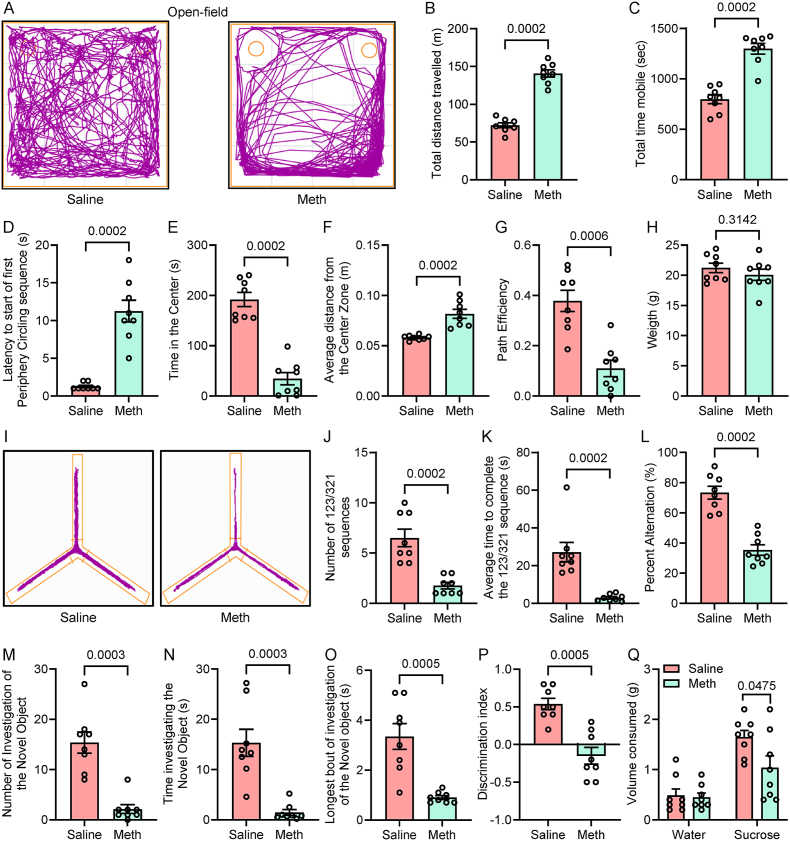
Effect of methamphetamine on the behavioral deficits in wildtype mice. Effect of methamphetamine on the behavioral deficits in wildtype mice. (A) Representative tracks from the open-field test performed in saline and methamphetamine-administered mice. Bar graphs showing the quantification of total distance traveled (B), total time mobile (C), latency to the start of the first periphery circling sequence (D), time spent in the center zone (E), average distance from the center zone (F), path efficiency (G), and weight (H), in mice administered with saline and methamphetamine. (I) Representative track from Y maze and bar graphs showing the quantification of number of 123/321 sequences (J), average time to complete the 123/321 sequence (K), Percent alternation (L) in saline and methamphetamine-administered mice. Bar graphs showing quantification of number of investigations of the novel object (M), time investigating the novel object (N), longest bout of investigation of the novel object (O), discrimination index (P), and volume consumed (Q), in saline and methamphetamine-administered mice. Statistical significance was determined using an unpaired Student's t-test.

To investigate whether NLRP6 inflammasome mediated changes contribute to methamphetamine-induced behavioral deficits, the open-field test was conducted. This test assesses anxiety-related behavior and locomotor activity by placing mice in a novel, unprotected environment. Representative movement tracks from the open-field test are presented in Fig. 6A. Methamphetamine-administered mice exhibited reduced mobility in the center of the arena compared to saline-administered controls, indicative of increased anxiety-like behavior.

Methamphetamine-administered mice also showed significant changes in locomotor activity and anxiety-related parameters. Specifically, total distance traveled (Fig. 6B) and total time spent mobile (Fig. 6C) were significantly increased, increased in methamphetamine-administered mice compared to saline controls, indicating hyperactivity. Methamphetamine-administered mice also demonstrated a shorter latency to initiate the first periphery circling sequence (Fig. 6D) and a higher average distance from the center zone (Fig. 6F), indicating a preference for the periphery and avoidance of the center zone, consistent with anxiety-like behavior. Interestingly, the time spent in the center zone was significantly lower in methamphetamine-administered mice compared to saline controls (Fig. 6E), further supporting an anxiety-like behavioral phenotype. In addition, the path efficiency (Fig. 6G) was significantly reduced in methamphetamine-administered mice, indicating disruptions in navigational strategies alongside hyperactivity and anxiety-related behaviors. Despite these behavioral changes, body weights were comparable between the methamphetamine-administered and saline-treated groups (Fig. 6H). These findings indicate that methamphetamine administration induces pronounced behavioral deficits, characterized by increased locomotion, anxiety-like behavior, and impaired navigational efficiency, which may be linked to NLRP6 inflammasome activation.

To further determine the effects of methamphetamine on spatial and working memory, we performed the Y maze and novel object recognition tests. Following training, all saline- and methamphetamine-administered mice underwent probe testing to assess memory performance. In the Y maze experiments, we analyzed the number and time required to complete 123/321 sequences, as well as the percentage of spontaneous alternations, comparing saline- and methamphetamine-administered mice. Representative heatmap tracks of the Y maze illustrate the mobility patterns of saline- and methamphetamine-administered mice throughout the experiment (Fig. 6I). Interestingly, methamphetamine-administered mice exhibited a significant decrease in the number of 123/321 sequences (Fig. 6J), the average time to complete a 123/321 sequence (Fig. 6K), and the percentage of spontaneous alternations (Fig. 6L) compared to the saline-treated group. These findings suggest impairments in spatial and working memory in methamphetamine-administered mice.

In the novel object recognition test, we evaluated the number of novel object investigations, the time spent investigating the novel object, and the longest bout of investigation in saline- and methamphetamine-administered mice. As anticipated, methamphetamine-administered mice exhibited a significant reduction in the number of novel object investigations (Fig. 6M), the time spent investigating the novel object (Fig. 6N), the longest bout of investigation (Fig. 6O), and the discrimination index (Fig. 6P) compared to saline-treated mice. These findings suggest that methamphetamine administration has negative effects on spatial and working memory, further emphasizing significant alterations in sequence pattern behavior and cognitive performance.

We also performed a sucrose preference test to assess depression-like behavior in the mice. Following training, both saline- and methamphetamine-administered mice were fasted overnight and then presented with a choice between regular water and a 1 % sucrose solution. For this experiment, we measured the weight differences between the start and end of the test phase and recorded the consumption of regular water and 1 % sucrose in grams. Interestingly, methamphetamine-administered mice showed a significant decrease in sucrose consumption compared to the saline-treated group, while water consumption remained similar between the two groups (Fig. 6Q). These findings suggest that methamphetamine administration may induce anhedonia, a hallmark of depression-like behavior.

### 3.7 Ex vivo validation of NLRP6 inflammasome signaling-mediated pyroptosis in adult astrocytes isolated from the frontal cortex of brain tissues from methamphetamine-administered mice

**Fig. 7:**
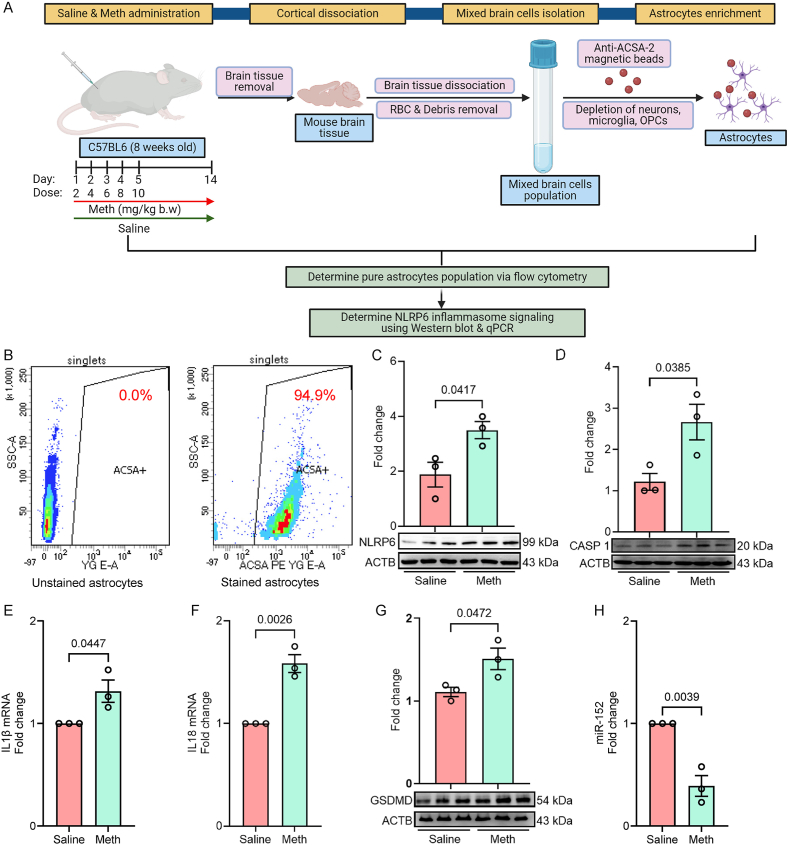
Validation of NLRP6 inflammasome signaling in *ex vivo* isolated adult astrocytes from saline- and methamphetamine-administered mice. (A) Schematic diagram of *ex vivo* adult astrocyte isolation from the mouse cortex using the ACSA-2 antibody. (B) Representative flow cytometry analysis showing astrocyte purity using the ACSA-2+ marker. Representative Western blot images and quantification of NLRP6 (C), cleaved caspase-1 (D), IL1β mRNA (E), IL18 mRNA (F), GSDMD (G), and miR-152 (H) in adult astrocytes isolated from the cortical brain regions of saline- and methamphetamine-administered wild-type mice. Statistical significance was determined using an unpaired Student's t-test. [Created using Biorender].

Following the observed increase in NLRP6 inflammasome activation and astrocyte activation in the brain tissues of methamphetamine-administered mice, we conducted an *ex vivo* study using adult astrocytes isolated from the cortical region of mice administered with saline and methamphetamine. This approach enabled us to specifically evaluate astrocyte activation and NLRP6 inflammasome signaling in isolation, independent of other brain cell types. The *ex vivo* isolated astrocytes provided a direct and focused assessment of the effects of methamphetamine on astrocytic activation and inflammasome signaling in wild-type mice. As shown in Fig. 7A, the astrocyte isolation process was optimized to yield clean, highly enriched populations suitable for subsequent analyses. Adult strocytes were isolated from the frontal cortex of methamphetamine and slaine administered adult mice, and their purity was confirmed via ACSA-2+ marker staining, which demonstrated a high purity of 94.9 % (Fig. 7B). This ensured that the observed effects were specific to astrocytes.

*Ex vivo* analysis also revealed a significant increase in the expression of NLRP6 inflammasome markers in astrocytes isolated from methamphetamine-administered mice. Notably, NLRP6 expression was markedly elevated (Fig. 7C), accompanied by increased caspase-1 cleavage (Fig. 7D), which confirmed inflammasome activation. Furthermore, the proinflammatory cytokines IL1β mRNA (Fig. 7E) and IL18 mRNA (Fig. 7F) were significantly upregulated, along with increased expression of GSDMD (Fig. 7G) indicating active pyroptotic signaling. Interestingly, astrocytes isolated from methamphetamine-administered mice showed a significant decrease in miR-152 expression (Fig. 7H), further implicating its involvement in regulating inflammasome activity. These *ex vivo* findings further validate the role of NLRP6 inflammasome signaling in methamphetamine-induced pyroptosis and astrocyte activation, corroborating the observations made *in vitro* and *in vivo*.

## 4 Discussion

Our study demonstrates that methamphetamine induces astrocyte activation and promotes neuroinflammation through NLRP6 inflammasome-dependent pyroptosis, mediated by the miR-152-NLRP6 axis. This is the first study to establish a direct link between methamphetamine exposure and the activation of NLRP6 inflammasome in astrocytes, providing novel insights into the molecular mechanisms underlying methamphetamine-induced neurotoxicity. We found that methamphetamine exacerbates proinflammatory responses by promoting NLRP6 oligomerization, caspase-1 activation, and gasdermin D cleavage, all of which culminate in pyroptotic cell death. These findings extend previous knowledge of methamphetamine-induced neuroinflammation by identifying the specific inflammasome pathways involved and their regulatory mechanisms. Importantly, our data highlight the role of miR-152 in modulating NLRP6 expression, suggesting a potential epigenetic mechanism that could be therapeutically targeted to mitigate astrocytic pyroptosis and the ensuing neuroinflammation. Together, these findings advance our understanding of the effects of methamphetamine on astrocytic function and immune homeostasis within the CNS and provide a foundation for future therapeutic strategies targeting inflammasome signaling in substance abuse-related neuroinflammation.

Methamphetamine has been reported to stimulate glial cell activation and disrupt CNS immune homeostasis, suggesting a link between methamphetamine-induced neurotoxicity and the neuroinflammatory response, wherein astrocytes play a central role [67,68]. Supportive studies have shown increased glial cell activation in the brains of methamphetamine abusers, as well as heightened expression of cellular organelle-mediated death pathways in methamphetamine-administered rats [69,70]. Furthermore, *in vivo* data indicate the involvement of noradrenergic brain regions, such as the medial basal forebrain, hippocampus, and prefrontal cortex, as evidenced by gliosis following methamphetamine administration [[71], [72], [73]]. Interestingly, a binge methamphetamine dosing regimen in wildtype mice revealed early astrocyte activation in the striatum, a key region of the dopaminergic pathways, which persisted for up to fourteen days, linking methamphetamine-induced neuroinflammation and neurotoxicity [12]. Complementary evidence from single-cell RNA sequencing of methamphetamine-exposed cerebral organoids demonstrated unique CNS cell type-specific morphological and phenotypic changes, along with robust transcriptional and proinflammatory responses [17]. Earlier studies also highlight astrocyte activation and migration involving the autocrine release of proinflammatory cytokines, underscoring the importance of cell-to-cell interactions in methamphetamine toxicity [74]. Additionally, receptors such as trace amine-associated receptor 1 and sigma-1 receptor have been implicated in methamphetamine-induced astrocytic responses [11,68,75]. Despite these findings, the precise molecular mechanisms linking astrocyte activation, neuroinflammation, and methamphetamine toxicity remain inadequately explored, emphasizing the significance of our current study.

There are at least two primary mechanisms proposed for methamphetamine-induced neuropathology: direct injury and bystander injury [9,76,77]. Direct injury refers to methamphetamine causing direct neuronal damage, while bystander injury involves methamphetamine exerting neuronal toxicity via inflammatory processes. Although the literature suggests both mechanisms could coexist, the bystander theory aligns more with methamphetamine-mediated astrocyte NLRP6 inflammasome-dependent pyroptosis. A recent study demonstrated that methamphetamine exposure mediated by astrocyte-microglia crosstalk induces glutamate release from astrocytes, leading to microglial activation, neuroinflammation, and impaired risk assessment behavior in mice [9]. Additionally, factors such as proinflammatory cytokine release and cell death in astrocytes contribute to neurotoxic effects, ultimately resulting in neuronal death and dysfunction [[78], [79], [80], [81]]. Methamphetamine has been shown to induce apoptosis and cell death in the brains of addicts and human cerebral organoids [17,82]. While previous studies indicated that methamphetamine induces necroptotic and apoptotic protein expression, causing cytokine excretion in other cell types [83], our findings highlight the essential role of pyroptosis in methamphetamine-induced astrocyte toxicity. This study also demonstrated that disulfiram, a pyroptosis inhibitor, provides maximum protection against methamphetamine-induced cell death compared to necroptosis or pan-caspase inhibitors. Notably, in a macaques study, *Macaca mulatta* pretreated with disulfiram (5.6 mg/kg) showed significantly reduced methamphetamine self-administration [84]. Based on these findings, we explored the molecular mechanisms involved in methamphetamine-induced astrocyte pyroptosis.

Herein, we, for the first time, demonstrated that NLRP6 inflammasome is critical for methamphetamine-mediated astrocyte activation driving the proinflammatory programmed cell death, pyroptosis. Given the essential role of innate immunity in the detection and counteracting of infectious agents and sterile insults, identifying the functions of innate immune sensors such as NLRP6 is fundamental to understanding health and disease [38,[85], [86], [87]]. While the specific triggers inducing NLRP6 inflammasome signaling in neuroinflammatory-related disease have not been well characterized [88], we found the significant expression of NLRP6, ASC oligomerization, cleaved caspase 1, proinflammatory cytokines release (IL1β and IL18), GSDMD oligomerization and pyroptosis in methamphetamine-exposed astrocytes. Also, this study showed increased expression of NLRP6 inflammasome signaling mediators and astrocyte activation in the frontal cortex, striatum, and hippocampus of methamphetamine-administered mice. These findings are consistent with the severe grey matter deficits observed in the cingulate, limbic, and paralimbic cortices and smaller hippocampal volumes observed in methamphetamine abusers [7,89]. Interestingly, we did not observe any significant difference in the expression of NLRP3 in methamphetamine-exposed mouse primary astrocytes. This is supported by an earlier investigation wherein higher concentrations of methamphetamine failed to increase NLRP3 expression [10]. Since astrocyte activation has dual effects on directly damaging neuronal function and other CNS cells via proinflammatory cytokine secretion [16,90,91], this study connects the role of methamphetamine-induced neuroinflammation with astrocyte activation. A previous study also demonstrated that the co-expression of NLRP6 with ASC leads to synergistic activation of NF-κB and the recruitment of PYPAF5 to punctate cytoplasmic structures [92], further supporting its involvement in pathways that may influence GFAP expression i.e. astrocyte activation.

Our results also demonstrated that miR-152 regulates methamphetamine-mediate NLRP6 inflammasome-dependent pyroptosis. In the miScript miRNA array data, we identified miR-152 as being significantly downregulated in methamphetamine-exposed mouse primary astrocytes when compared with control cells. Since miR-152 has not been explored in the context of methamphetamine-induced toxicity before, we focused on its dysregulation in the pathogenesis of methamphetamine-mediated NLRP6 inflammasome signaling. Consistent with the miScript miRNA array results, we observed a marked decrease of miR-152 expression in the qPCR assay of methamphetamine-exposed mouse primary astrocytes. Moreover, we observed a negative correlation between miR-152 overexpression and methamphetamine-induced NLRP6 inflammasome-dependent pyroptosis in mouse primary astrocytes, suggestive of its anti-inflammatory properties. In addition, our results identified a marked decrease in miR-152 expression in methamphetamine-administered mice. miR-152 has been reported to be evolutionarily conserved, possesses an identical seed sequence in different species, and is critical in the inhibition of oxidative stress, inflammation, and apoptosis in several diseases [[93], [94], [95]]. Interestingly, a recent investigation demonstrated that miR-152 conferred protection against intracerebral hemorrhage-mediated neuroinflammation and brain injury by inhibiting thioredoxin-interacting protein-induced inflammasome activation in both *in vivo* and *in vitro* models [96]. Collectively, these results further suggest that dysregulation of miR-152 is involved in the progression of methamphetamine-induced NLRP6 inflammasome-dependent pyroptosis, thus contributing to the overall induction of methamphetamine-mediated neuroinflammation.

Several epidemiological studies have shown the persistence of anxiety, psychotic, cognitive impairment, and depression-like behavior as the main symptoms associated with methamphetamine misuse [[97], [98], [99]]. In addition, astrocyte activation and the persistent production and secretion of proinflammatory cytokines have also been linked to neurodegeneration observed in methamphetamine-induced disorders [67,68,[100], [101], [102]]. Various studies have also highlighted inflammation as a probable mechanism in methamphetamine-induced depression and anxiety [[103], [104], [105]]. Additionally, earlier investigations showed that sigma-1 receptor antagonists protected wildtype mice against methamphetamine-induced neurotoxicity by blocking glial cell activation and proinflammatory cytokines expression, thus underscoring the critical role of methamphetamine-mediated neuroinflammation [75,106].

Our behavioral data also support the neurotoxic effects of methamphetamine. Mice exhibited significant anxiety-like behaviors, including increased total distance traveled, increased time spent mobile, delayed initiation of peripheral circling, and reduced time in the center zone during the open-field test [23,107]. These observations are consistent with anxiety-like behaviors reported in methamphetamine-abusing patients and in preclinical models [103,108,109]. Additionally, we observed a notable reduction in percentage alternation, the number of novel object investigations, time spent investigating the novel object, and the longest bout of investigation in the Y maze and novel object tests, respectively. Existing data have implicated significant cognitive impairment and learning difficulties in methamphetamine users, which corroborates our findings [110,111]. Interestingly, our results revealed that methamphetamine mediates stress-induced anhedonia, a critical symptom of depression observed in mice. This result has also been reported in a community cohort of people who smoke methamphetamine, but abstinence from the drug led to improvements in both clinical and social outcomes [112,113]. Short-term methamphetamine administration in our study resulted in a clear progression of toxicity, highlighting the importance of inflammation as an early feature of methamphetamine-induced neurodegeneration [101,114,115]. Although our findings align with the observed behavioral deficits, the specific contribution of NLRP6 inflammasome signaling to cognitive impairment and anxiety-like behavior requires further exploration.

**Fig. 8:**
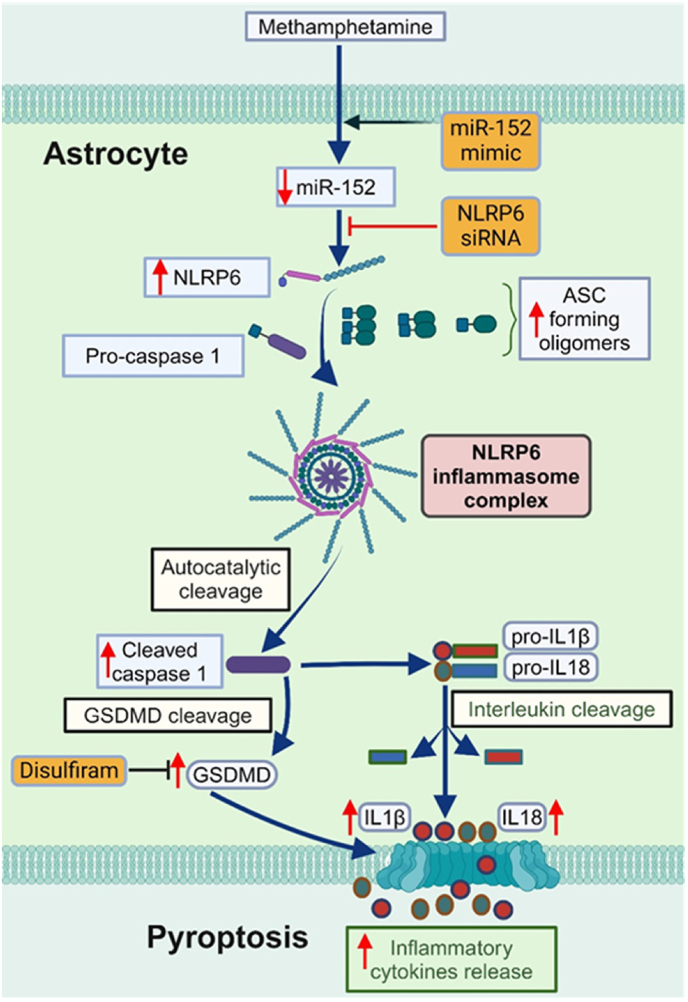
Schematic representation of the proposed mechanism underlying methamphetamine-mediated NLRP6 inflammasome-dependent pyroptosis in astrocytes. Methamphetamine exposure activates the NLRP6 inflammasome by modulating miR-152 in astrocytes, leading to the cleavage of caspase-1 and the subsequent maturation and release of proinflammatory cytokines IL1β and IL18. It also triggers pyroptosis, characterized by the formation of pores in the plasma membrane via GSDMD. This schematic highlights the central role of NLRP6 in mediating these inflammatory and cell death processes, suggesting it as a potential target for therapeutic intervention in methamphetamine-induced neurotoxicity. [Created using Biorender].

In summary, our study demonstrates that methamphetamine induces astrocyte NLRP6 inflammasome-dependent pyroptosis and proinflammatory cytokine release, with miRNA-152 playing a regulatory role. Increased NLRP6 expression drives inflammasome activation, leading to enhanced proinflammatory cytokine secretion and astrocyte activation, which contribute to neuroinflammation (Fig. 8). However, the absence of specific pharmacological inhibitors for NLRP6 limits the full biochemical characterization of this pathway. Targeting the NLRP6 inflammasome or its downstream mediators could offer a promising therapeutic strategy to mitigate methamphetamine-induced neuroinflammation. The use of NLRP6 knockout (KO) mice could further advance our understanding of the therapeutic potential of NLRP6 in addressing methamphetamine-induced neuroinflammatory signatures [62,116]. Although the use of NLRP6 KO mice presents challenges due to its whole-body knockout design, astrocyte-specific knockdown approaches, such as AAV-GFAP-NLRP6 shRNA vectors, could provide more precise insights into the role of NLRP6 in methamphetamine-mediated neuroinflammation. Additionally, methamphetamine-induced addiction and withdrawal remain central challenges in treating methamphetamine abuse. Understanding the role of NLRP6 inflammasome signaling in addiction and withdrawal warrants further investigation. Future studies should also explore the involvement of Toll-like receptors in methamphetamine-induced NLRP6 signaling, as these receptors are known to initiate adaptive immune responses. Given the anti-inflammatory and neuroprotective properties of miR-152, miR-based therapy should also be proposed for translational application in methamphetamine-mediated neuroinflammation in both animal and human studies.

## CRediT authorship contribution statement

**Abiola Oladapo:** Writing – review & editing, Writing – original draft, Visualization, Validation, Methodology, Investigation, Formal analysis, Data curation. **Muthukumar Kannan:** Writing – review & editing, Validation, Methodology. **Uma Maheswari Deshetty:** Writing – review & editing, Validation, Methodology. **Seema Singh:** Writing – review & editing, Validation, Methodology. **Shilpa Buch:** Writing – review & editing, Supervision, Resources, Conceptualization. **Palsamy Periyasamy:** Writing – review & editing, Visualization, Supervision, Resources, Methodology, Investigation, Funding acquisition, Formal analysis, Data curation, Conceptualization.

## Declaration of competing interest

The authors declare that they have no known competing financial interests or personal relationships that could have appeared to influence the work reported in this paper.

## Data Availability

Data will be made available on request.
