# Supplementary material for: Methamphetamine-mediated astrocytic pyroptosis and neuroinflammation involves miR-152–NLRP6 inflammasome signaling axis
Source: Redox Biol. 2025 Jan 25;80:103517. doi: 10.1016/j.redox.2025.103517 (PMC11810843; doi:10.1016/j.redox.2025.103517)
Supplement: Multimedia component 1 [file mmc1.pdf]

Methamphetamine-mediated astrocytic pyroptosis and neuroinflammation involves miR-152–NLRP6 inflammasome signaling axis

Abiola Oladapo, Muthukumar Kannan, Uma Maheswari Deshetty, Seema Singh, Shilpa Buch, and Palsamy Periyasamy*

Department of Pharmacology and Experimental Neuroscience, University of Nebraska Medical Center, Omaha, NE, 68198-5880, USA

* Corresponding author at: Department of Pharmacology and Experimental Neuroscience, University of Nebraska Medical Center, Omaha, NE, 68198-5880, USA

E-mail address: palsamy.periyasamy@unmc.edu (P.Periyasamy)

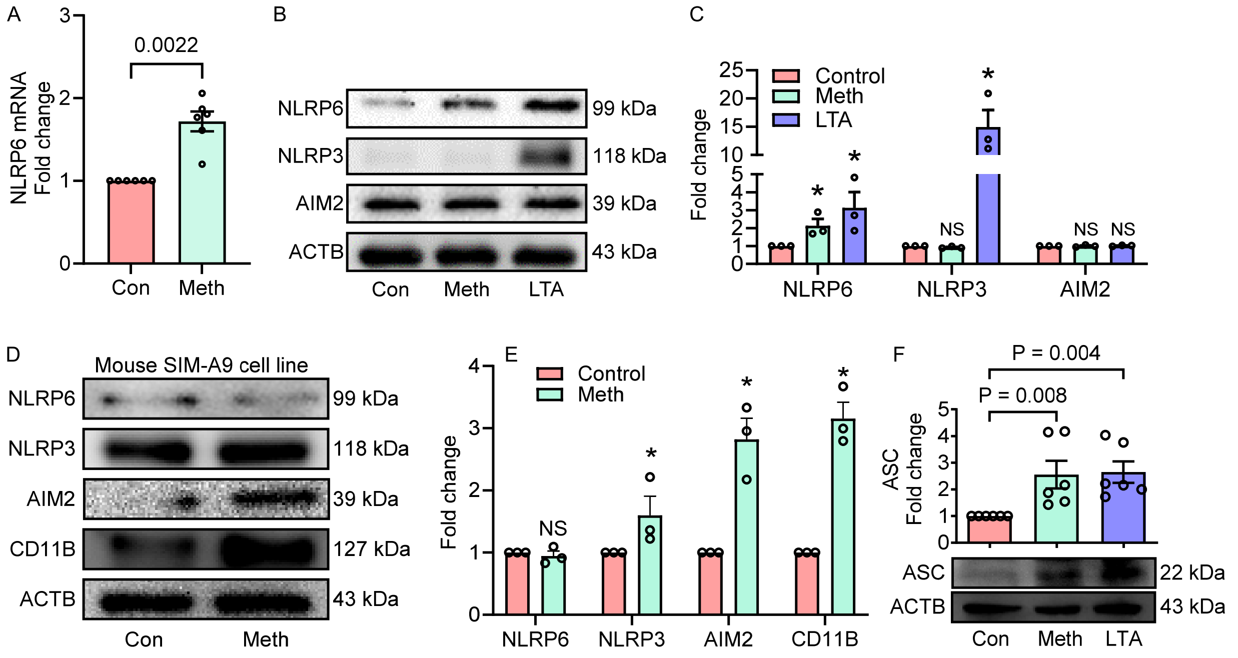

Fig. S1: Expression profile of inflammasome proteins in methamphetamine-exposed mouse primary astrocytes and mouse SIM-A9 cell line. (A) qPCR showing the mRNA expression of NLRP6 in mouse primary astrocytes exposed to 50 μM methamphetamine for 24 hours (N=6). (B) Representative western blot and quantification showing the expression of NLRP6, NLRP3, and AIM2 in mouse primary astrocytes exposed to 50 μM methamphetamine for 24 hours (N=3). (D and E) Representative western blot and quantification showing the expression of NLRP6, NLRP3, AIM2 and CD11B in mosue SIM A9 cell line exposed to 50 μM methamphetamine for 24 hours (N=3). (F) Representative western blot and quantification showing the expression of ASC in mouse primary astrocytes exposed to 50 μM methamphetamine for 24 hours (N=6). β-actin was used as an internal control for all experiments, and the data are presented as mean ± SEM. LTA (5 ng/ml) was used as a positive control. Statistical significance was determined using an unpaired Student's t-test. *P < 0.05 versus control.

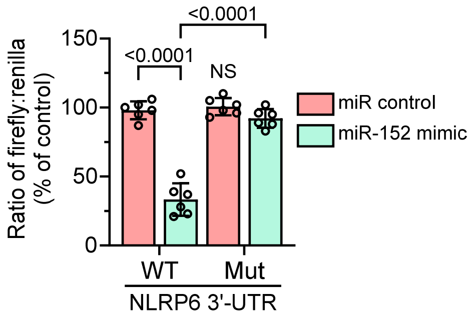

Fig. S2: Dual luciferase assay confirms that miR-152 targets the 3′-UTR of NLRP6. HEK293T cells were co-transfected with a pmirGLO dual luciferase reporter plasmid containing either the wild-type (WT) NLRP6 3′-UTR or a mutated (Mut) NLRP6 3′-UTR, along with miR-152 mimic. Data are presented as mean ± SEM. Statistical significance was determined using a nonparametric Kruskal–Wallis one-way ANOVA followed by Dunn's post hoc test. *P < 0.05 compared to the control.

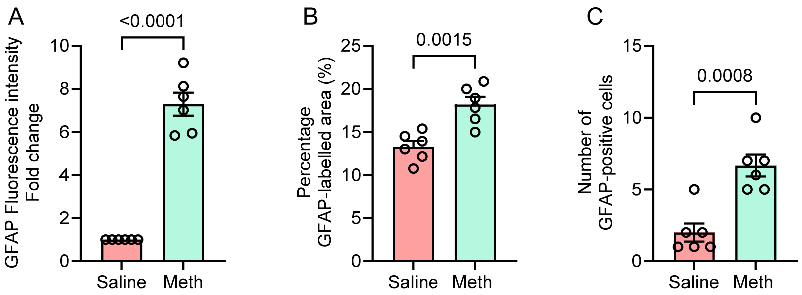

Fig. S3: Methamphetamine-induced astrocyte activation in the hippocampus of wildtype mice. (A) Bar graph showing the quantification of GFAP fluorescence staining intensity, (B) percentage of GFAP-labeled area, and (C) number of GFAP-positive cells in the hippocampus of wild-type mice administered methamphetamine or saline. Data are presented as mean ± SEM. Statistical significance was determined using an unpaired Student's t-test. *P < 0.05 versus saline.

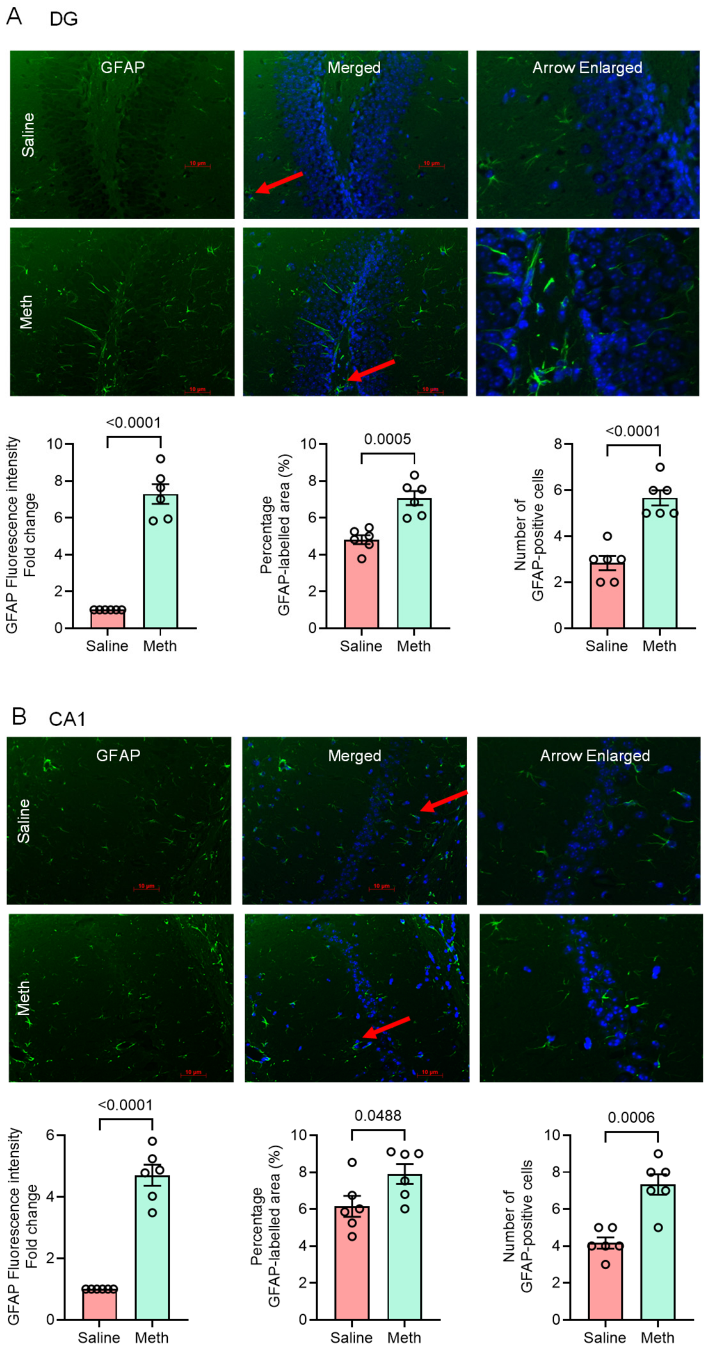

Fig. S4A-B: Methamphetamine-induced astrocyte activation in the subregions of the hippocampus in wild-type mice. Representative immunohistochemistry images and quantification showing GFAP expression in the hippocampal subregions: DG (A), and CA1 (B) of wild-type mice administered methamphetamine or saline (scale bar: 10 μm). Data are presented as mean ± SEM. Statistical significance was determined using an unpaired Student's t-test. *$P < 0.05$ versus saline.

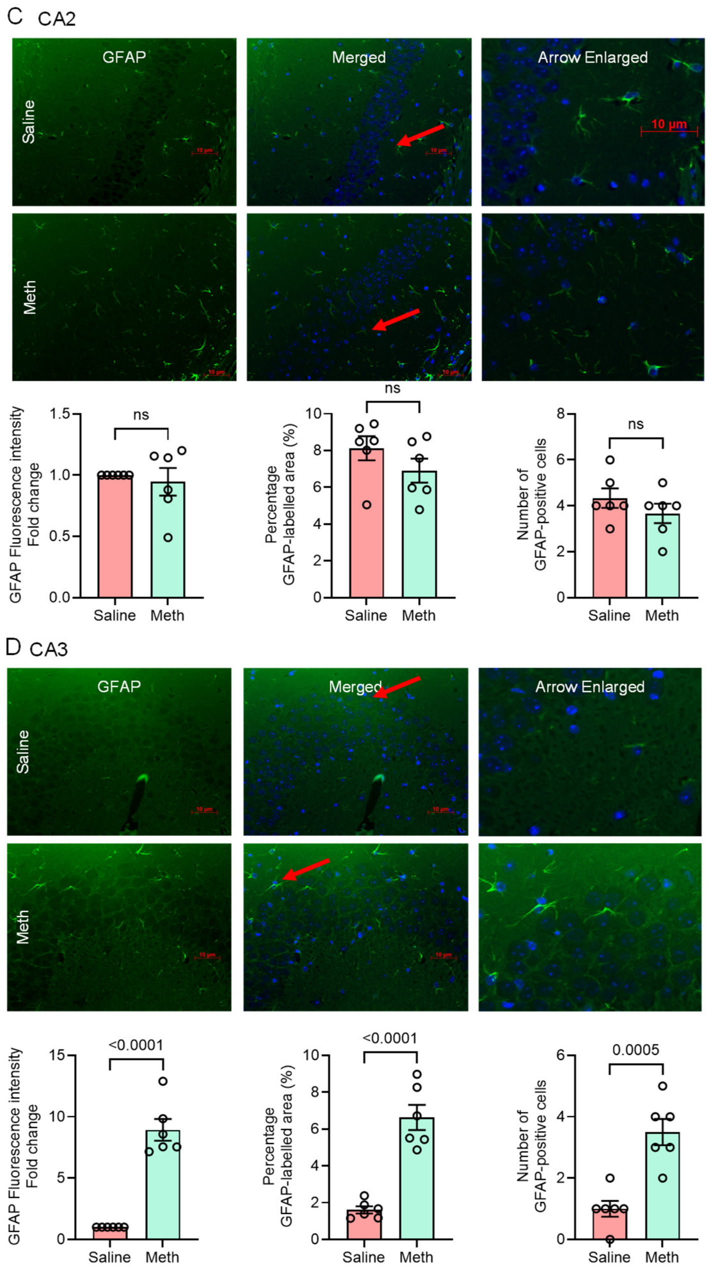

Fig. S4C-D: Methamphetamine-induced astrocyte activation in the subregions of the hippocampus in wild-type mice. Representative immunohistochemistry images and quantification showing GFAP expression in the hippocampal subregions: CA2 (C), and CA3 (D) of wild-type mice administered methamphetamine or saline (scale bar: 10 μm). Data are presented as mean ± SEM. Statistical significance was determined using an unpaired Student's t-test. *P < 0.05 versus saline.

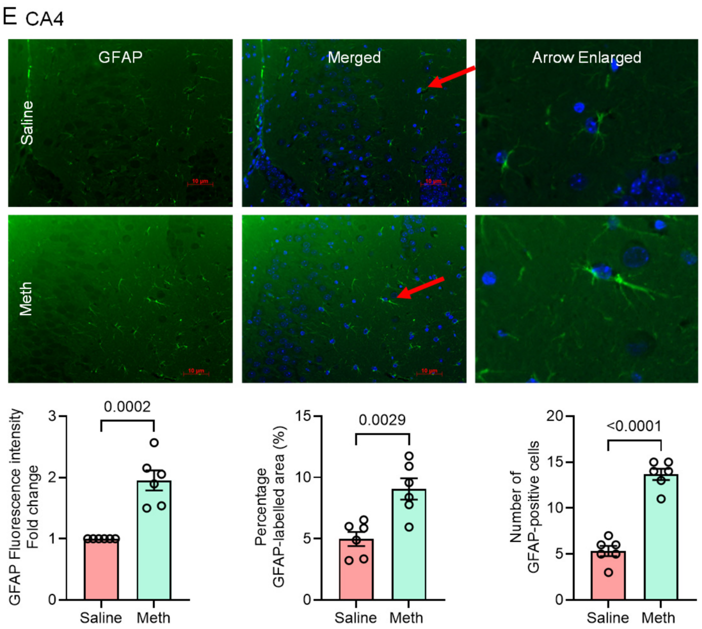

Fig. S4E: Methamphetamine-induced astrocyte activation in the subregions of the hippocampus in wild-type mice. Representative immunohistochemistry images and quantification showing GFAP expression in the hippocampal subregions: CA4 (E) of wild-type mice administered methamphetamine or saline (scale bar: 10 μm). Data are presented as mean ± SEM. Statistical significance was determined using an unpaired Student's t-test. $^*P < 0.05$ versus saline.

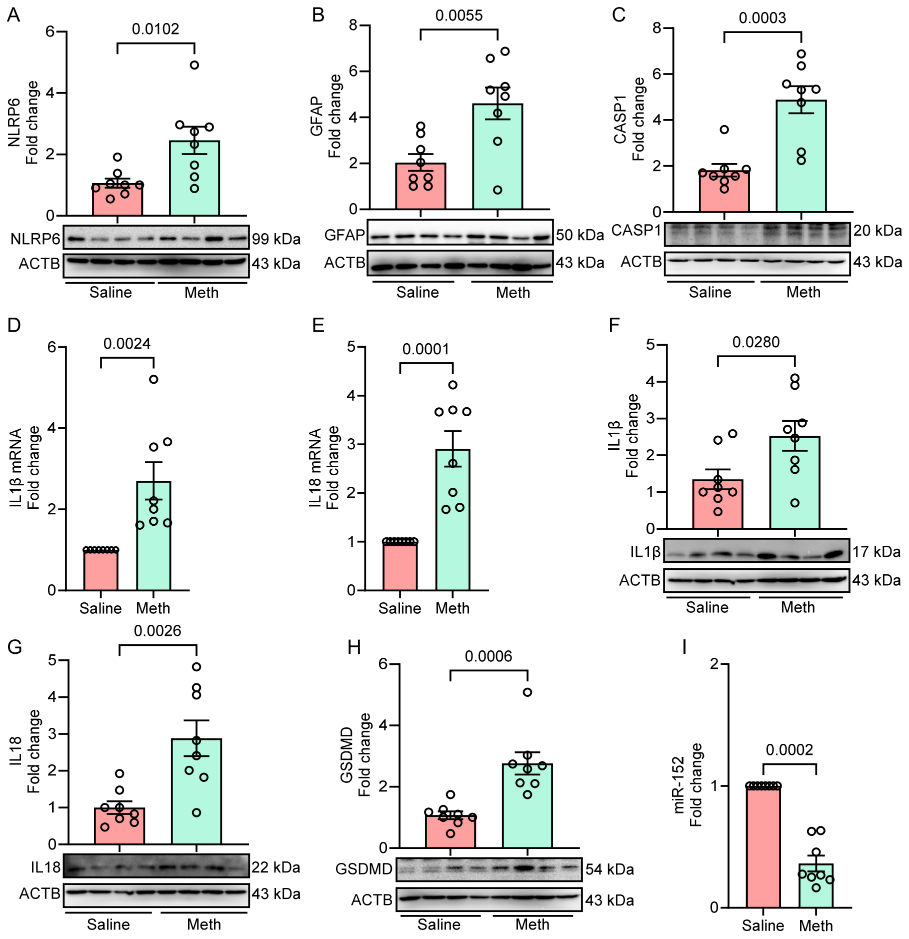

Fig. S5: Methamphetamine-induced NLRP6 inflammasome signaling and astrocyte activation in the frontal cortices of wildtype mice. Representative western blot images of (A) NLRP6, (B) GFAP, and (C) cleaved caspase-1 in the frontal cortices of wildtype mice administered with methamphetamine or saline. qPCR analysis showing the mRNA expression of (D) IL1β and (E) IL18 in the frontal cortices of wildtype mice administered with methamphetamine or saline. Representative western blot images of (F) IL1β, (G) IL18, and (H) GSDMD in the frontal cortices of wildtype mice administered with methamphetamine or saline. (I) qPCR analysis showing the expression of miR-152 in the frontal cortices of wildtype mice administered with methamphetamine or saline. β-actin was also used as an endogenous control. Data are presented as mean ± SEM. Statistical significance was determined using an unpaired Student's t-test. $^{*}P < 0.05$ versus saline. Representative western blot images are shown for n=4 per group, as it was not feasible to run all samples on a single blot. However, quantification was performed for n=8 per group using two separate rounds of western blotting.

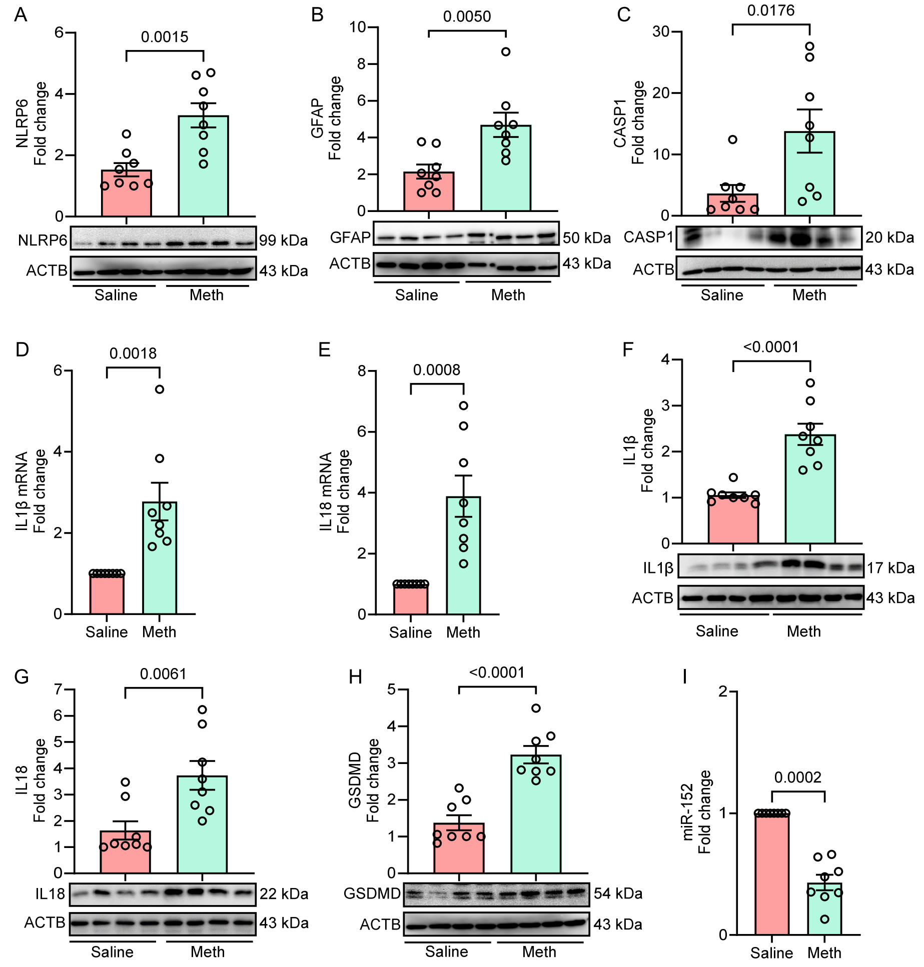

Fig. S6: Methamphetamine-induced NLRP6 inflammasome signaling and astrocyte activation in the striatum of wildtype mice. Representative western blot images of (A) NLRP6, (B) GFAP, and (C) cleaved caspase-1 in the striatum of wildtype mice administered with methamphetamine or saline. qPCR analysis showing the mRNA expression of (D) IL1β and (E) IL18 in the striatum of wildtype mice administered with methamphetamine or saline. Representative western blot images of (F) IL1β, (G) IL18, and (H) GSDMD in the striatum of wildtype mice administered with methamphetamine or saline. (I) qPCR analysis showing the expression of miR-152 in the striatum of wildtype mice administered with methamphetamine or saline. β-actin was also used as an endogenous control. Data are presented as mean ± SEM. Statistical significance was determined using an unpaired Student's t-test. *$P < 0.05$ versus saline. Representative western blot images are shown for n=4 per group, as it was not feasible to run all samples on a single blot. However, quantification was performed for n=8 per group using two separate rounds of western blotting.
